# Targeted K‐Edge Nanoprobes From Praseodymium and Hafnium for Ratiometric Tracking of Dual Biomarkers using Spectral Photon Counting CT

**DOI:** 10.1002/advs.202408408

**Published:** 2024-10-07

**Authors:** Nivetha Gunaseelan, Parikshit Moitra, Pranay Saha, Teresa Aditya, Mahdieh Moghiseh, Kevin Jonker, Steven Gieseg, Anthony Butler, Fadia Kamal, Dipanjan Pan

**Affiliations:** ^1^ Huck Institutes of the Life Sciences Department(s) of Biomedical Engineering Nuclear Engineering Materials Science and Engineering The Pennsylvania State University 101 Huck Life Sciences Building University Park PA 16802 USA; ^2^ Department of Pediatrics Centre for Blood Oxygen Transport & Hemostasis University of Maryland Baltimore School of Medicine Baltimore Maryland 21201 USA; ^3^ Department of Chemical & Biochemical Engineering University of Maryland Baltimore County Baltimore Maryland 21250 USA; ^4^ MARS Bioimaging Limited 68 Saint Asaph Street, Christchurch Central City Christchurch 8011 New Zealand; ^5^ School of Biological Sciences University of Canterbury Private Bag 4800 Christchurch 8041 New Zealand; ^6^ Department of Pathology and Biomedical Science University of Otago 2 Riccarton Avenue Christchurch 8011 New Zealand; ^7^ Center for Orthopaedic Research and Translational Science Department of Orthopaedics and Rehabilitation Penn State College of Medicine The Pennsylvania State University Hershey PA 17033 USA; ^8^ Present address: Department of Chemical Sciences Indian Institute of Science Education and Research Berhampur Berhampur Odisha 760003 India

**Keywords:** hafnium nanoparticles, K‐edge imaging, osteoarthritis tracking, praseodymium nanoparticles, quantitative imaging, spectral photon counting computed tomography

## Abstract

Utilizing metal nanoprobes with unique K‐edge identities to visualize complementary biological activities simultaneously can provide valuable information about complex biological processes. This study describes the design and preparation of an innovative pair of K‐edge metal nanoprobes and demonstrates the feasibility of their simultaneous quantitative detection using spectral photon‐counting computed tomography (SPCCT). Glycosaminoglycan (GAG) capped nanoparticles (ca. 15–20 nm) targeting two distinct components of the cartilage tissue, namely, aggrecan (acan) and aggrecanase (acanase) are designed and synthesized. These targeted nanoparticles comprised of praseodymium (Pr) and hafnium (Hf), with well‐separated K‐edge energies, enable simultaneous and ratiometric imaging of dual biomarkers in cartilage tissue. Following extensive physico‐chemical characterization of the ligand‐targeted particles, the feasibility of homing dual biomarkers in vitro is demonstrated. The material discrimination and simultaneous quantification of these targeted particles are also achieved and corroborated with inductively coupled plasmon spectroscopy. For the first time, the use of praseodymium is reported as a contrast agent for SPCCT imaging and demonstrates the ability to pair it with hafnium nanoprobes for multicontrast imaging of diseases. Importantly, the potential for ratiometric molecular imaging and tracking of osteoarthritis (OA) progression is shown with SPCCT K‐edge based imaging approach.

## Graphical abstract

Schematic showing workflow of spectral photon counting CT with Medipix 3RX detector to detect and quantify dual nanoprobes from praseodymium and hafnium metals. The nanoprobe is carefully chosen due to their unique K‐edge identities that enable maximum material discrimination from bone, soft tissue, and water in biological samples as seen in the multicolor 3D rendering of the human bone joint.

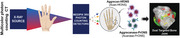

## 1 Introduction

When complementary biological activities are visualized simultaneously, the complexity of disease pathology can be better understood. The use of dual K‐edge metal nanoprobes may allow simultaneous detection of these activities through the targeting of two biomarkers with interdependent expression profiles, where each probe provides specific molecular information, and their combined visualization allows for improved diagnosis.^[^ ^1^, ^2^ ^]^ Although conventional CT scans are widely used as a first‐line diagnostic tool due to their ability to quickly and non‐invasively provide detailed images of internal structures, they are plagued by limitations when it comes to multicontrast imaging due to low spatial resolution and poor material separation.^[^ ^3^ ^]^ Therefore, several efforts have been made in the field of multicontrast imaging using other modalities such as MRI,^[^ ^4^ ^]^ photoacoustic imaging,^[^ ^5^ ^]^ multi‐isotope nuclear imaging modalities^[^ ^6^, ^7^ ^]^ as well as dual‐energy CT.^[^ ^8^ ^]^ However, the recent emergence of SPCCT imaging has shown to provide a universal quantitative set of “colors” that can be used to decompose materials and correct artifacts in conventional CT scans.^[^ ^9^, ^10^ ^]^ A prime benefit of SPCCT is its ability to separate more than two materials based on their unique K‐edge identities.^[^ ^11^, ^12^, ^13^ ^]^ Several literature reports have shown the potential of SPCCT in revolutionizing biological imaging.^[^ ^14^, ^15^, ^16^, ^17^ ^]^ SPCCT has shown improved spatial resolution compared to conventional CT and dual‐energy CT with a pixel size of 90 microns that enables improved soft tissue contrast at extremely low radiation dosage.^[^ ^18^ ^]^ Its clinical relevance has significantly increased, particularly with the FDA 510(k) clearance of the Siemens Naeotom Alpha, the first photon‐counting CT scanner developed for clinical use.^[^ ^19^, ^20^ ^]^ Although the modality has immense potential, its molecular imaging capability for clinical applications is still largely unexplored. This high‐resolution, K‐edge based imaging approach could potentially be used to image multiple biological targets thus enabling disease progression tracking with time by obtaining quantitative ratiometric information from biomarker expression profiles. The approach can be particularly beneficial in skeletal disease diagnosis since the progression of cartilage degradation is highly variable among patients and the ratiometric information from complementary markers could provide crucial information about the stage of the disease.

**Figure 1:**
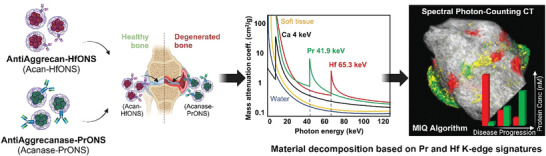
Schematic representation showing synthesized GAG capped‐hafnium and praseodymium metal nanospheres for ratiometric tracking of complementary OA biomarkers in joint cartilage. Healthy and degenerated joints were imaged using SPCCT and material decomposition (MD) was applied based on the unique K‐edge signatures of the two metals. 3D rendering shows a treated human metacarpophalangeal joint imaged using SPCCT and the nanomolar (nM) binding concentration quantified using the Material Identification and Quantification (MIQ) algorithm.

To achieve this, SPCCT requires highly sensitive, specific, and accurate imaging probes made of metals with appropriately placed K‐edge energies within the CT bandwidth. Metal nanospheres have long been used as contrast agents for medical imaging due to their strong X‐ray attenuation and tunable properties such as size, shape, and surface characteristics.^[^ ^21^ ^]^ Several reports have indicated that hafnium‐based contrast agents provide optimal X‐ray contrast efficiency compared to iodine and gadolinium due to its high atomic number (Hf; *Z* = 72) and an ideally‐positioned K‐edge (65.3 keV).^[^ ^22^, ^23^ ^]^ Alongside hafnium, in this report, we have identified praseodymium (*Z* = 59), a rare lanthanide metal with K‐edge, 41.9 keV that could potentially be paired with hafnium as an exogenous contrast agent for dual probe imaging using SPCCT. Although there have been recent efforts to synthesize lanthanide metal‐based nanoparticles for imaging,^[^ ^24^ ^]^ to our knowledge, praseodymium‐based nanoparticles have not been explored as a potential contrast agent for CT imaging. Hence, we have chosen the unique combination of hafnium (Hf, K‐edge: 65.3 keV) and praseodymium (Pr, K‐edge: 41.9 keV) as a K‐edge metal nano pair designed to concurrently target two complementary biomarkers of interest. This metal pair falls into two different SPCCT energy bins and hence provides minimal signal overlap leading to highly efficient material discrimination. (**Figure** **1**) The two complementary biomarkers with potential prognostic value that were identified were aggrecan and aggrecanase. Aggrecan, a core protein in articular cartilage, is essential for cartilage resistance to compression and shock absorption. Aggrecanase (ADAMTS5) plays a significant role in cartilage degeneration and OA disease progression and is upregulated in human OA chondrocytes.^[^ ^25^, ^26^ ^]^ It cleaves the aggrecan core protein, causing its irreversible degradation which disrupts the balance between aggrecan synthesis and breakdown leading to cartilage degeneration.^[^ ^27^ ^]^ Degenerated cartilage has reduced shock absorption and increased friction, which triggers inflammation and exacerbates OA development and progression. Healthy articular cartilage function requires high concentration of aggrecan, sulfation, and large proteoglycan aggregate formation. However, in OA joints, these properties are impaired due to the cleavage of the IGD domain in the aggrecan protein core.^[^ ^28^, ^29^ ^]^ Understanding aggrecan and aggrecanase roles in OA has led to potential therapeutic and diagnostic strategies, such as imaging aggrecan concentrations,^[^ ^30^ ^]^ designing aggrecanase inhibitors,^[^ ^31^ ^]^ and therapies promoting aggrecan synthesis.^[^ ^32^ ^]^ Therefore, we have targeted these biomarkers, with inversely dependent expression levels that provide a strong correlation with OA severity, using a unique pair of K‐edge nanoprobes quantified with SPCCT imaging.

Herein, we successfully synthesized and physico‐chemically characterized two novel contrast probes, praseodymium oxide nanosphere targeted to aggrecanase (acanase‐PrONS) and hafnium oxide nanosphere targeted to aggrecan (acan‐HfONS) for multicontrast imaging of cartilage using SPCCT. The colloidal nanoparticles were stabilized using chondroitin sulfate (CS), a sulfated GAG that provides resistance to compression of cartilage. Currently, there are no known literature reports of CS GAG mediated targeting agent, and we, for the first time, demonstrate ratiometric K‐edge imaging using dual CS GAG – directed metal nanoprobes and SPCCT imaging.

## 2 Results and Discussion

### 2.1 Synthesis and Physico‐Chemical Characterization of Praseodymium and Hafnium Nanospheres Capped with GAG and Target Ligands

**Figure 2:**
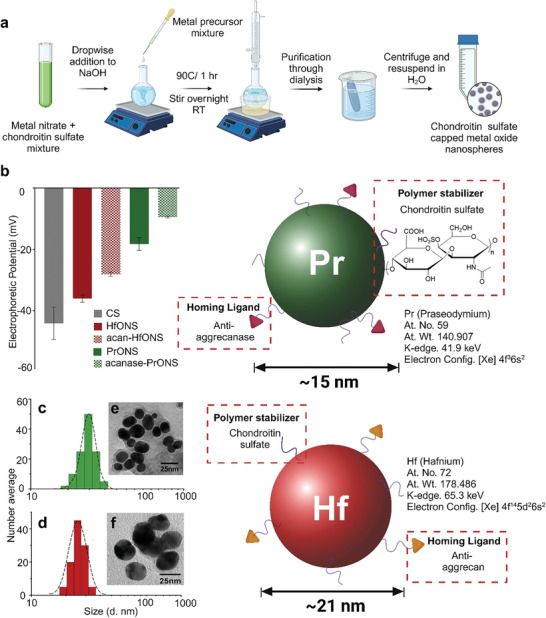
a) Schematic representation of nanoprobe synthesis process. Spherical nanoparticles capped with CS polymer were synthesized by nitrate reduction of metal salt and surface functionalized with homing ligands via EDC mediated coupling; Characterization of the synthesized nanoprobes b) Electrophoretic zeta potential values showing an increase in potential of nontargeted particles versus pure CS and a further increase in potential with successful ligand functionalization; DLS measurements of c) PrONS d) HfONS showing hydrodynamic size distribution of the capped particles, inset TEM images showing anhydrous diameter of e) PrONS and f) HfONS, scale bar = 25 nm.

**Figure 3:**
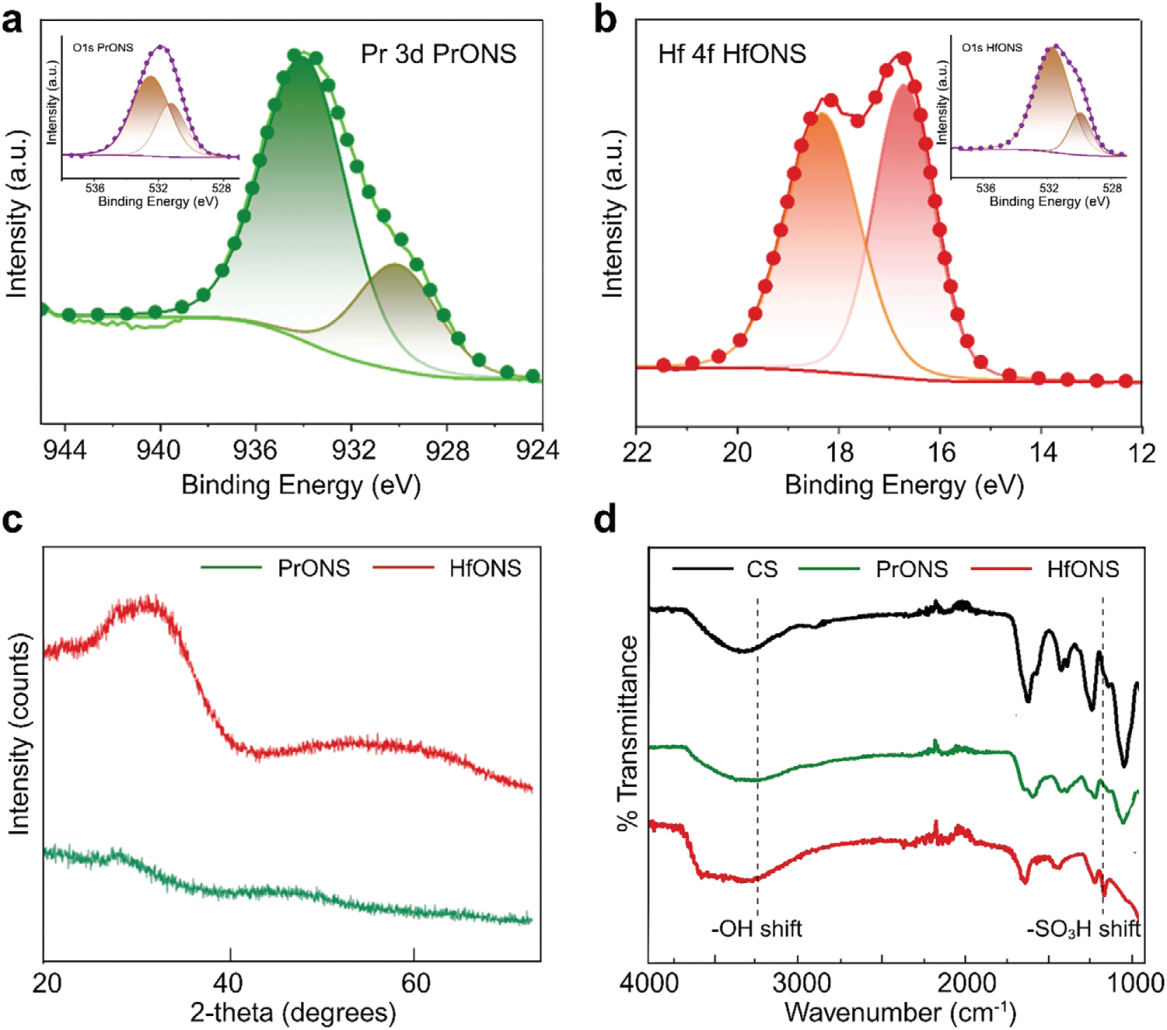
a) XPS data showing a) deconvoluted Pr 3d5/2 spectra of PrONS; b) deconvoluted Hf 4f spectra of HfONS; insets showing binding energies of electrons from O1s orbitals; c) XRD spectra of HfONS and PrONS showing amorphous structure of synthesized particles; d) attenuated total reflectance FTIR spectra of CS capped PrONS and HfONS versus pure CS.

Spherical praseodymium oxide and hafnium oxide nanoparticles were synthesized via a solvothermal nitrate reduction method from praseodymium and hafnium nitrate precursors.^[^ ^24^ ^]^ This one‐pot synthesis involved dropwise addition of the mixture of metal nitrate and polymer capping agent at a predetermined ratio to a base solution, resulting in nanosphere formation with surface abundant carboxyl groups. (**Figure** **2**a) This surface chemistry enabled the functionalization of the targeting ligands, aggrecanase, and aggrecan antibodies to the respective metal nanospheres via 1‐ethyl‐3‐(3‐dimethylaminopropyl) carbodiimide (EDC), N‐hydroxysuccinimide (NHS) coupling method. The number averaged hydrodynamic diameter of the resulting nanospheres, PrONS and HfONS, were measured to be 27 ± 2 nm and 29 ± 3 nm respectively (Figure 2c,d) from dynamic light scattering (DLS). The transmission electron microscopy (TEM) images show the formation of well‐dispersed spherical particles, and the average anhydrous diameter of the synthesized PrONS and HfONS was calculated to be 15 ± 3 nm and 21 ± 1 nm respectively with polymer capping (Figure 2e,f). TEM and Energy‐dispersive X‐ray spectroscopy (EDS) (Figure S1, Supporting Information) show the presence of Pr and Hf metal in the nanoparticles along with the distribution of sulfur due to the presence of the CS outer layer as expected. Thermogravimetric analysis (TGA) was done to observe the difference in mass loss trend between pure CS and the synthesized nanospheres. Figure S2 (Supporting Information) plots show the TGA and the corresponding derivative curves of the decomposition of CS. PrONS and HfONS were allowed to equilibrate in nitrogen at a heating rate of 10 °C min^−1^ up to 600 °C. The decomposition event for CS shows a peak ≈270 °C with a minor peak ≈100 °C for surface absorbed water molecules while the metal decomposition occurred in multiple mass loss steps due to the presence of Pr and Hf oxide. Subsequently, X‐ray diffraction (XRD) measurements of both PrONS and HfONS were collected to understand the crystal structure of the synthesized particles and the results are shown in (**Figure** **3**c). The spectrum for both particles shows an amorphous structure with no defined crystal peaks. The successful polymer capping of the metal nanospheres and surface functionalization with the target ligands was characterized by physico‐chemical methods. Electrophoretic ζ‐potentials of the polymer‐coated PrONS and HfONS were measured to be −18 ± 2 mV and −36 ± 2 mV, respectively which increased from the standalone ζ‐ potential of CS (−44 ± 5 mV). The negative potentials indicate the dense abundance of ‐COOH groups on the surface of the particles and the high electrophoretic potential values demonstrate their colloidal stability. Successful conjugation of anti‐aggrecan and anti‐aggrecanase antibodies was confirmed by the observed ζ‐potential shifts, −10 ± 1 mV for acanase‐PrONS and −28 ± 1 mV for acan‐HfONS upon surface ligand functionalization. (Figure 2b)

Further analysis of the functional groups was obtained by Fourier transform infrared spectroscopy (FTIR) (Figure 3d). The peaks at 3354 cm^−1^ were attributed to the ─OH asymmetric stretching and the peaks observed at 1614 and 1225 cm^−1^ were attributed to the C═O stretching vibration in the carbonyl group and the S═O stretching vibration in the sulfonic group respectively. In the spectrum of CS, these peaks were preserved while we observed peak shifts for these bonds in the nanoparticles possibly due to the coordination of Hf and Pr metal ions with the hydroxyl, carbonyl, and sulfonyl oxygen. X‐ray photoelectron spectroscopy (XPS) measurements were used to analyze the presence of C, N, O, S, Pr, and Hf in the synthesized particles to reveal their chemical composition (Figure S3, Supporting Information). The XPS spectra for PrONS show three prominent features of C1s peak at 289.70, 288.07, 285.92 and 284.32 eV that can be assigned to O─C═O, N─C═O/C─OSO_3_, C─O/C─N and C─C/C─H bonds respectively, N1s peak at 399.81 eV, O1s peaks at 532.67 eV for the adsorbed oxygen and at 531.36 eV for metal‐oxygen bond of Pr─O. S2p peaks of sulfur in PrONS can be ascribed to S2p1/2 at 170.33 eV and S2p3/2 at 168.85 eV. The Pr spectrum showed a core level peak at binding energy 933.61 eV and a shake‐off satellite peak (929.1 eV) at the lower binding energy side of the metal peak. The core peak was identified as the Pr^3+^ 3d5/2 component of Pr3d which confirms the oxide nanoparticle structure as Pr_2_O_3._ (Figure 3a; Figure S4, Supporting Information) Similarly, the HfONS XPS spectra also reveal three prominent features of C1s peak at 289.03, 288.00 286.04 and 284.47 eV for the above‐mentioned carbon groups, N1s peak at 399.64 eV, O1s peaks for similar kind of bonding at 531.68 eV and 529.92 eV. S2p peaks were observed ≈170.23 eV for S2p1/2 and 168.89 eV for S2p3/2. The Hf spectrum reveals clear symmetric double peaks at 18.32 eV and 16.71 eV for 4f5/2 and 4f7/2, respectively identified as the Hf^4+^ 4f7/2 component of Hf 4f which confirms the oxide nanoparticle structure as HfO_2_ (Figure 3b; Figure S4, Supporting Information). Table S1 (Supporting Information) shows the individual atomic percentages of the elements detected in both the particles. The XPS data thus agreed well with the FTIR results, further revealing the presence of surface binding sulfonic and carboxyl functional groups of PrONS and HfONS as expected. Finally, Raman spectroscopy measurements were recorded to confirm Pr and Hf metal presence and the successful polymer capping of the particles (Figure S5a, Supporting Information). HfO_2_ exhibited peaks ≈167 and 176 cm^−1^ whereas the Pr_2_O_3_ Raman peaks were observed ≈434 and 445 cm^−1^ showing the presence of metal oxides in the synthesized particles.^[^ ^33^ ^]^ A dominant CS peak was observed ≈1200 cm^−1^ due to the vibration of SO_3_^−^ asymmetric stretching and a strong peak ≈1000 cm^−1^ was observed due to the OSO_3_^−^ symmetrical stretching. These CS specific peaks were also observed in the Raman spectra of the nanospheres indicating successful polymer surface conjugation. Figure S5b,c (Supporting Information) plots show the Raman spectra for acan‐HfONS and acanase‐PrONS that confirm successful functionalization of the antibodies on the particle surface.

We further explored the percent binding of aggrecanase and aggrecan antibodies with PrONS and HfONS respectively to develop targeted contrast agents with optimal binding efficiency. To determine the conjugated antibody concentrations, Bradford assay was performed, separating the conjugated and free antibodies post conjugation process. The antibody concentration of the supernatant was measured using predetermined standard curves and the conjugated antibody concentration was calculated by subtracting the concentration in the supernatant from the initial antibody concentration.^[^ ^34^, ^35^, ^36^ ^]^ The percentage conjugation provided the optimal balance between achieving sufficient antibody attachment for target binding and maintaining nanoparticle stability to avoid non‐specific binding in biological samples. This assay showed an optimum of 1% surface conjugation of the respective antibodies to the nanosphere surface.

### 2.2 Standardization of SPCCT Protocol for Sensitive Detection and Delineation of Hf and Pr Nanoprobes

**Figure 4:**
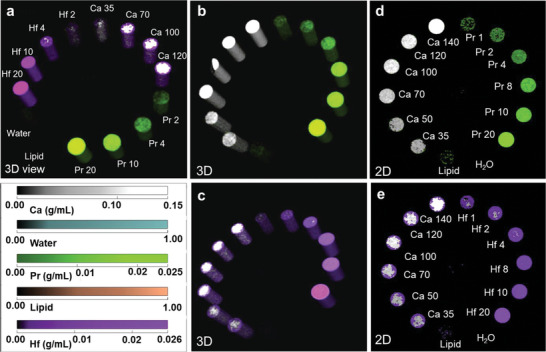
a) 3D reconstructed view of the MARS material phantom loaded with varying concentrations of praseodymium and hafnium with lipid and water for protocol development/optimization and material decomposition in biological samples. The concentration ranges used for both praseodymium and hafnium were 2, 4, 10 and 20 mg mL^−1^. Materials have been identified by the MIQ algorithm and color coded using the MARS Vision software praseodymium (green), hafnium (purple), calcium (white) to show the accuracy in material decomposition and exact location of the materials in the phantom. b) 3D reconstructed views of a phantom scan with increased concentration ranges of praseodymium and calcium for improved material identification; c) 3D reconstructed views of a phantom scan with increased concentration ranges of hafnium and calcium for improved material identification; d) 2D view of the Pr phantom scan with color coded regions of Pr (green) versus Ca (white) showing minimal cross talk and concentration range used Pr (1, 2, 4, 8, 10 and 20 mg mL^−1^) and Ca (35, 50, 70, 100, 120, 140 mg mL^−1^); e) 2D view of the Hf phantom scan with color coded regions of Hf (purple) versus Ca (white) and concentration range used Hf (1, 2, 4, 8, 10 and 20 mg mL^−1^) and Ca (35, 50, 70, 100, 120, 140 mg mL^−1^).

Ideal SPCCT agents must combine the visibility of contrast agents but also have the K‐edge energies well separated to avoid overlapping. A phantom study was designed to assess the feasibility of using Pr and Hf simultaneously for SPCCT imaging. **Figure** **4**a shows the phantom designed for simultaneous detection of Pr and Hf. The MARS Microlab 5×120 small bore SPCCT scanner (MARS Bioimaging Ltd., New Zealand), with an advanced cadmium zinc telluride (CZT)‐assembled Medipix3RX detector was used to conduct these phantom studies. This study was conducted to optimize a unique protocol to accurately discriminate PrONS and HfONS in biological samples. The protocol was initially tailored to identify Pr with high sensitivity and accuracy. It was necessary to scan two different phantom combinations in order to develop a protocol to successfully identify PrONS and reduce material crosstalk between them. The first scan included a PMMA (polymethyl methacrylate) phantom loaded with 2 mL Eppendorf PCR tubes containing PrONS solutions of various concentrations (1, 2, 4, 8, 10, 20 mg mL^−1^), CaCl_2_ (Ca) vials (35, 50, 70, 100, 120, 140 mg mL^−1^), water, and lipid as shown in Figure 4b,d. The second PMMA phantom was loaded with increased concentration range (0.2, 0.5, 1, 2, 4, 8, 10, 20, 40, 60 mg mL^−1^) of PrONS standard, water, and lipid to increase the sensitivity and detection range of the system (Figures S7 and S8a, Supporting Information). Similarly, an individual phantom scan for HfONS and Ca was also done to accurately detect Hf with minimal cross talk with Ca as shown in Figure 4c,e. With the capability of the system to simultaneously sample up to four energy bins plus an aberration counter with an energy resolution of ≈2.5 keV, the energy thresholds were set strategically at 30, 45, 60, and 76 keV in the Charge Summing Mode (CSM) to cover the K‐edge values of both Pr and Hf. Pixel masking was applied prior to scanning to remove noisy pixels, including non‐functional, high and low‐sensitive pixels and the calibration phantoms scans were reconstructed using the MARS Algebraic Reconstruction Technique (mART).^[^ ^37^, ^38^, ^39^, ^40^ ^]^ Linear attenuation (LA) and standard deviation for each concentration and material in the phantoms were quantified and converted to Hounsfield Units (HU) by manually selecting a region of interest (ROI) over each vial. Linear regression analysis was used to determine the relationship between HU and material concentration to obtain a linear response curve.^[^ ^41^ ^]^ One axial slice was selected from each energy bin and the relationship between HU and the energy bin was evaluated graphically and the spectral response was recorded (Figure S8, Supporting Information). MIQ^[^ ^42^ ^]^ algorithm was employed using the MARS in‐house program to calculate the effective mass attenuation of each calibration vial and obtain the material image datasets of Pr, water, lipid, and Ca. Each material was accurately identified in the vials and their identification percentages were recorded as shown in Table S4 (Supporting Information). The linear attenuation and HU linearity curves show high R^2^ values for both Ca and Pr which indicate that the protocol is sensitive with accurate material identification and quantification. (Figure S8, Supporting Information) Similar material quantification was done for the phantom scan with extended Pr concentration range. (Table S2, Supporting Information) The protocol identified Pr concentrations higher than 0.5 mg mL^−1^ accurately. The measured specificity for even lower concentrations was 40% indicating a high enough confidence to detect concentrations lower than 1 mg mL^−1^ within biological samples. This thorough process enabled the successful identification of a Pr contrast agent that had not been explored for SPCCT previously. A similar process was followed for optimizing the protocol to identify Hf using HfONS loaded calibration phantom with varying concentrations (1, 2, 4, 8, 10, 20 mg mL^−1^) as shown in Figure 4c,e. Following the individual Pr and Hf calibrations, a final phantom scan was done using both the contrast agents to calibrate the system for concurrent detection of Pr and Hf with minimal cross talk between both materials. Figure 4a shows the 3D reconstructed image of the final phantom (Figure S6a, Supporting Information) with Pr and Hf in the concentration range of 2, 4, 10 and 20 mgmL^−1^ including Ca, water and lipid to mimic the biological samples. The color code shows the material decomposition applied by the MIQ algorithm to identify each material accurately. Figure S8 (Supporting Information) shows the linear and spectral response obtained from the PrONS calibration vials scanned with lipid, water and Ca across all the energy bins. As expected, the K‐edge effects for Pr and Hf were observed in the second (30‐45 keV) (Figures S8 and S9, Supporting Information) and fourth energy (60–75 keV) bins respectively due to their unique K‐edge energy values. The fitted linear lines demonstrate the linear response of the system (*R^2^* > 0.99), for both Pr (Figure S8d,e, Supporting Information) and Hf (Figure S9c,d, Supporting Information) which show the accurate identification of both materials simultaneously by the MARS scanner.

### 2.3 Design of Ex Vivo Human Hand Model Mimicking OA for the Evaluation of SPCCT Sensitivity and Accuracy in Dual Nanoprobe Quantification

**Figure 5:**
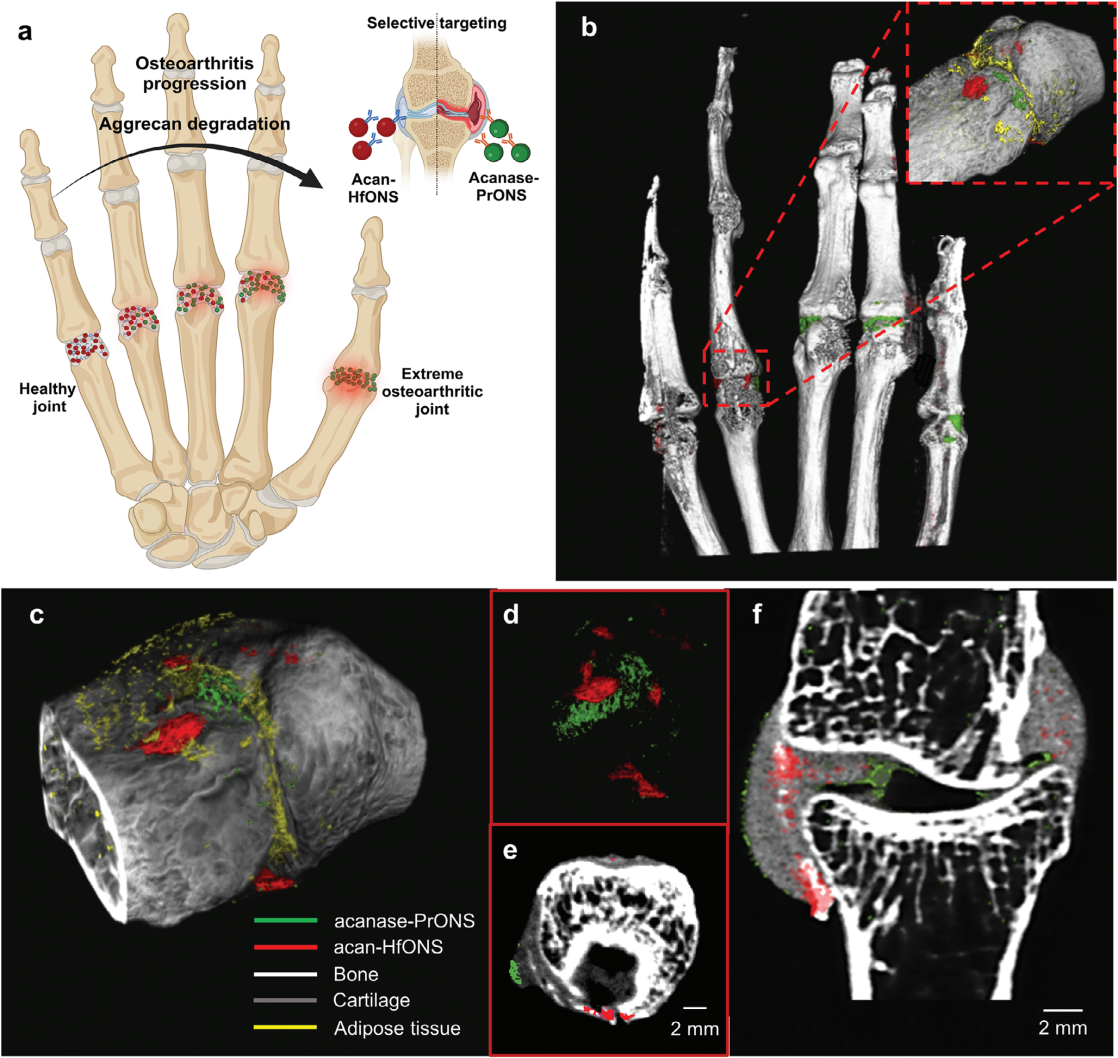
a) Schematic showing *ex vivo* model of OA progression; b) 3D reconstructed image of the human hand with bound nanoprobes visualized by MARS Vision; c) 3D representative image of dual targeting using PrONS and HfONS with MD applied; d) targeted acanase‐PrONS (green) and acan‐HfONS (red) material delineated and visualized in e) axial view f) coronal view of the treated bone joint; scale bar = 2 mm.

**Figure 6:**
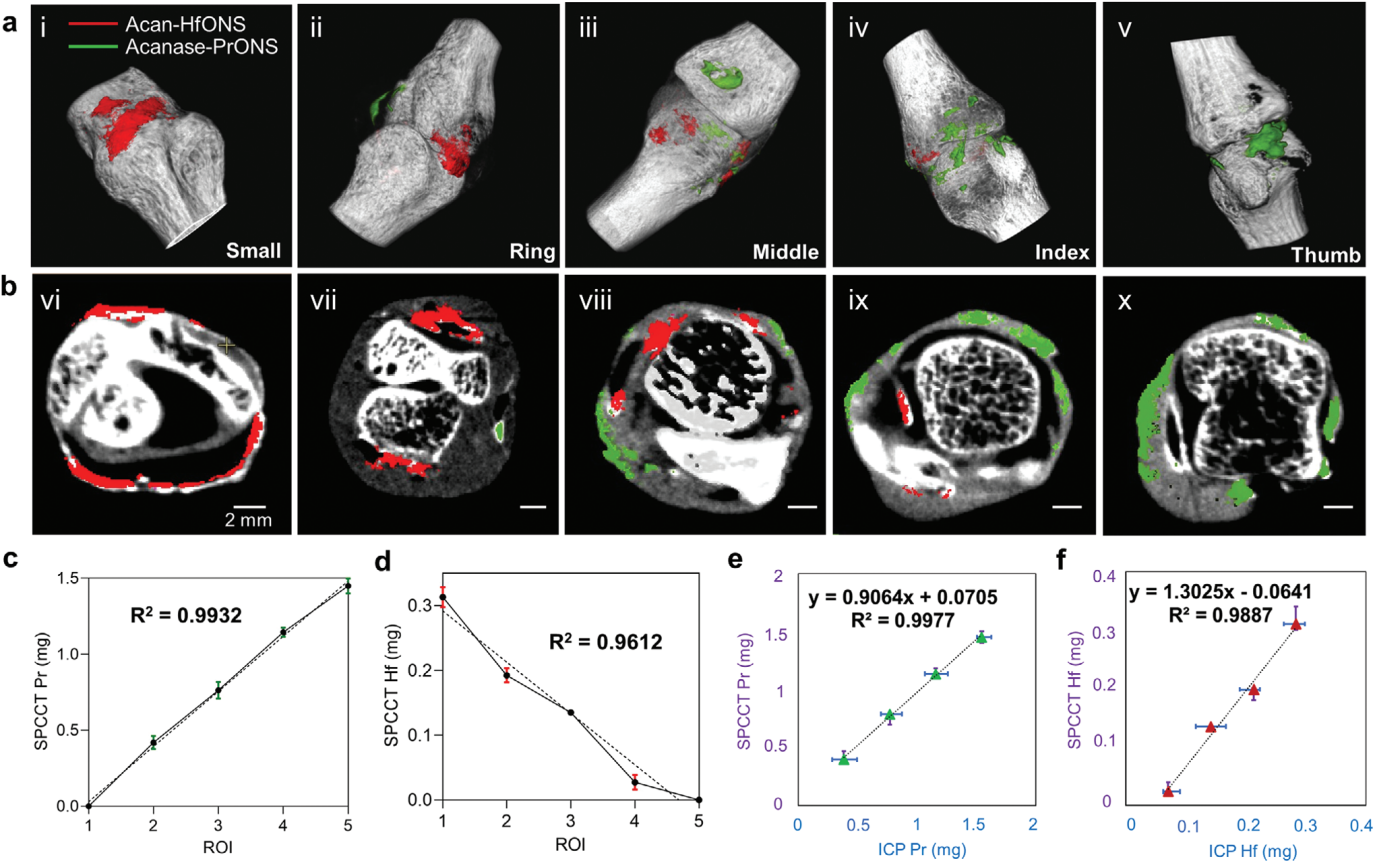
a) 3D reconstructed images of the individual joints showing selective accumulation of acanase‐PrONS and acan‐HfONS with OA progression from left to right; i–v show 3D views of each joint from small, ring, middle, index and thumb in the direction of increasing OA severity b) vi–x show the corresponding axial slices of the joints showing the varying ratios of nanoprobe binding; The color code hafnium (red) in the sample is differentiated from praseodymium (green) and bone (white) and cartilage (gray); Graphs showing linear change in metal concentration across the ROIs (regions of interest) detected by the MARS scanner; c) Aggrecanase targeted Pr metal concentration (mg) linearly increases with each joint (R^2^ = 0.99); d) Aggrecan targeted Hf metal concentration (mg) decreases linearly showing aggrecan degradation with disease severity (R^2^ = 0.96); (ROI joints 1‐ small, 2‐ ring, 3‐middle, 4‐index, 5‐thumb); Comparative analysis of two quantification methods (ICP‐OES versus SPCCT) to demonstrate the accuracy (%error) based on the metal content levels identified in individual bone joints. The two methods correlate with regression value e) R^2^ = 0.9977 for Pr metal content, f) R^2^ = 0.9887 for Hf metal content and show strong validity of SPCCT detection and quantification of multiple contrast agents. Data is presented as mean ± standard deviation (*N* = 3).

We explored the possibility of using these targeted metal nanoprobes as imaging agents in SPCCT for OA detection at varying stages of disease progression using human phalanges. For the *ex vivo* study, we devised multiple models of OA conditions in the human phalangeal joints to mimic the progression of OA severity (**Figure** **5**a). The acan‐HfONS and acanase‐PrONS were injected into the cartilage to mimic accumulation of targeted nanoprobes at varying capacities at different stages of OA severity. The small finger joint mimics a normal healthy joint with the highest aggrecan concentration followed by a gradual decrease of aggrecan levels from the small finger to the thumb joint. On the contrary, the aggrecanase concentration decreases from thumb joint to the small finger with the thumb joint representing severe OA condition with maximum aggrecan degradation. (Figure 5b) The accumulation of targeted PrONS and HfONS in the cartilage layer of each joint successfully simulates the aggrecanase‐mediated aggrecan degradation in varying capacities to mimic a realistic in vivo scenario. Figure 5c–f shows the scan images and material decomposition of a representative OA joint with targeted PrONS and HfONS binding. The human hand joints were scanned using the MARS MicroLab SPCCT scanner to simultaneously detect and quantify the signal received from both the targeted PrONS and HfONS (**Figure** **6**a,b). The total time for a single scan of the human hand was ≈40 min. The optimized Pr and Hf MARS scanning protocol from the initial phantom studies was used for the biological sample study. The scanner protocol was set at 110 kVp, 20 µA x‐ray tube current and 160 ms exposure time, to acquire a high statistic scan with 720 projections over 360° rotation maximizing the accuracy of detecting both nanoparticles, as well as 720 flat–field (open‐beam) projections to remove Poisson noise. A field of view (FOV) of 110 mm, with two camera translations, was large enough to cover the entire human hand sample. The optimized protocol (Table S3, Supporting Information) with strategically placed energy bin thresholds enabled maximizing the photon counts and contrast signal from the individual nanoprobes. The image reconstruction with applied material decomposition (MD) was carried out at 90 µm voxel size to achieve maximum resolution within reasonable time duration. Individual axial slices from the MD datasets post reconstruction were used to quantify the Pr and Hf metal concentrations at each joint and analyze the accuracy of identification for the biological samples. The 3D‐reconstructed images clearly show impressive material discrimination of the bound PrONS and HfONS due to their ideally placed K‐edge signatures while differentiating the contrast agents from the surrounding cartilage tissue and underlying bone.

**Figure** **6**c,d show the linear change in quantitative signals obtained from the bound acanase‐PrONS and acan‐HfONS at different joints due to the increasing disease severity which correlates with aggrecan degradation mediated by increased aggrecanase expression levels in the corresponding joint. The study successfully demonstrates the efficacy of using these metal nano pairs as potential exogenous contrast agents for quantitatively tracking dual biomarkers. The mapping of the elements and material quantification in the biological samples was done using the inbuilt MARS Vision software (Figure 6). The MIQ algorithm identified a linearly decreasing quantitative trend of aggrecan‐bound HfONS and an inversely dependent increasing trend for aggrecanase‐bound PrONS in the nanomolar (nM) concentration range from normal to severe OA joint as expected. Joints i (Normal), ii, iii, iv, v (Severe OA) were quantified at 8.12, 5.06, 3.54, 0.71, 0 nM of acan‐HfONS and 0, 58.04, 105.44, 158.37, 200.38 nM of acanase‐PrONS as shown in Figure 6c,d.

Both PrONS and HfONS provided unique attenuating characteristics ideal for simultaneous spectral CT imaging with minimal cross talk and high sensitivity and specificity. The metal content quantification by the MARS scanner was cross‐verified using inductively coupled plasma–optical emission spectroscopy (ICP‐OES) analysis where the nanoprobe samples were fully digested in aqua regia, to measure the individual metal concentrations in each joint. (**Table** **1**) The ICP calibration curves were used to interpolate the metal concentration of the diluted samples to the concentration present in the *ex vivo* human hand joints as shown in (Figure S10, Supporting Information) and correlate the measured concentrations with the quantified data obtained from the MARS SPCCT scanner as shown in Figure 6e,f. The high correlation between the SPCCT and ICP analysis proved the accuracy of the MARS scanner in detecting the two materials with high sensitivity and thus showed the potential of utilizing these contrast agents simultaneously for multicontrast imaging.

**Table 1:** Quantitative comparison of PrONS and HfONS average metal content in human phalangeal joints calculated by MARSVision versus ICP‐OES analysis of metal content ^a)^ in biological samples shows successful material identification and quantification of the nanoprobe binding in each joint. Data presented as mean ± standard deviation *(N = 3*).

ROI (Joint)	ICP Pr [nM]	ICP Hf [nM]	SPCCT Pr [nM]	SPCCT Hf [nM]
Small	0	7.36 ± 0.45	0	8.12 ± 0.39
Ring	53.62 ± 14.51	5.48 ± 0.42	58.04 ± 6.08	5.06 ± 0.29
Middle	107.65 ± 12.02	3.72 ± 0.66	105.44 ± 7.6	3.54 ± 0.03
Index	160.16 ± 13.13	1.97 ± 0.37	158.37 ± 4.15	0.71 ± 0.29
Thumb	214.89 ± 8.43	0	200.38 ± 6.91	0

^a)^ mg mL^−1^ concentrations have been converted to nanomolar (nM) for ICP and SPCCT quantification of absolute metal content.

### 2.4 Design of In Vitro Multilayer and 3D Spheroid Models Mimicking OA Progression for Evaluating Binding Efficacy of Hf and Pr Nanoprobes

**Figure 7:**
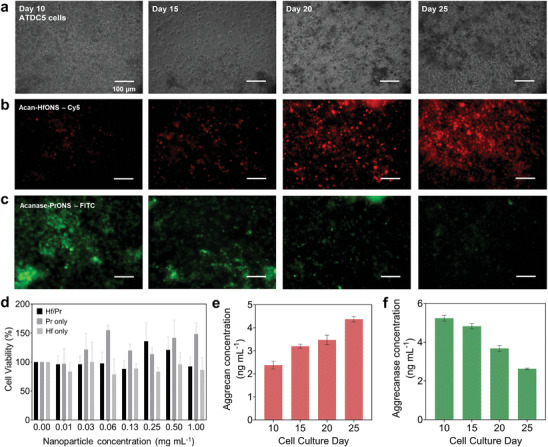
Selective binding of acanase‐PrONS and acan‐HfONS in vitro in ATDC5 chondrogenic cell line a) Brightfield images showing ATDC5 cell growth and viability at day 10, 15, 20, and 25 of culture. ECM mineralisation observed with an increase in the number of cell culture days. Fluorescence images of ATDC5 cells treated with targeted b) Acan‐HfONS color coded by Cy5 channel (red) c) Acanase‐PrONS color coded by FITC channel (green) throughout culture days 10 to 25, scale bar = 100 µm; d) MTT assay showing % cell viability versus treatment with targeted HfONS only, PrONS only and both HfONS / PrONS; Bar plots showing gradual e) increase in aggrecan GAG concentration (ng mL^−1^) f) decrease in aggrecanase concentration (ng mL^−1^), observed in ATDC5 cells with chondrogenesis at days −10, −15, −20, and −25 using ELISA for protein quantification. Data presented as mean ± standard deviation (*N* = 3).

Motivated by the ex vivo results, an in vitro chondrogenesis model was developed.^[^ ^43^ ^]^ This model was expected to evaluate the efficacy of these agents in detecting the target proteins and to understand the toxicity profiles of the synthesized nanoprobes within the cellular environment. A mouse teratocarcinoma cell line, ATDC5, was used for this study since these cells have the capacity to differentiate into chondrocyte‐like cells with a cartilage‐like environment. The ATDC5 in vitro study was aimed at understanding the differential particle uptake of acanase‐PrONS and acan‐HfONS based on the expression levels of aggrecanase and aggrecan. Generally, it is found that more aggrecan is produced in the extracellular matrix (ECM) of multi‐layer ATDC5 cell culture.^[^ ^44^ ^]^ Multiple sub cultures at different time points were treated with the targeted nanoprobes. We noticed that ATDC5 cells, incubated with both acan‐HfONS and acanase‐PrONS, showed varying levels of particle uptake due to the varying aggrecan and aggrecanase levels present in the cells and ECM at different stages of chondrocyte differentiation. The targeted PrONS were fluorescently tagged with FITC and targeted HfONS were tagged with Cy5 to visualize the differential uptake of both particles at 10‐, 15‐, 20‐, and 25‐day time points of cell culture using fluorescence microscopy. MTT assay was performed to determine the optimum concentration of the metal nanospheres that could be used to treat cells without toxicity. It is expected that because of the polymer surface coating, the toxicity of these particles will be minimal. The structural similarity between these particles in terms of size, shape, and surface coating allows similar internalization by the ATDC5 cells with minimal toxicity. **Figure** **7**d shows the percentage of relative cell viability calculated for cell groups treated with acanase‐PrONS, acan‐HfONS, and both particles simultaneously. The data is presented as mean ± standard deviation (*N* = 3) for each treatment group. We observed a slight decrease in cell viability when the cells were treated with both particles compared to the individual treatment groups. However, cell viability did not differ significantly between control and treatment groups, indicating that the polymer‐coated PrONS and HfONS are highly biocompatible. As the particles are non‐toxic, 0.25 mg mL^−1^ of each probe was chosen for the cell incubation studies, since this concentration yielded the best results in the MTT assay for cells treated with both particles. Figure 7b shows the gradual increase in Cy5 fluorescence indicating increased acan‐HfONS uptake due to elevated aggrecan levels during chondrogenesis differentiation. Aggrecan production starts at day 10 and gradually increases through days 15 to 20. At day 25, the ATDC5 cells show maximum aggrecan expression levels with high ECM development. Similarly, the FITC fluorescence in Figure 7c shows the gradual targeted uptake of acanase‐PrONS due to the decrease of aggrecanase in the cell line with increased aggrecan production from days 10 to 25. Similar cell culture batches were used to determine the increase in aggrecan levels at the same time points using enzyme‐linked immunosorbent (ELISA) assay. The assay showed a gradual increase in aggrecan concentration with time and maximum levels at day 25 of cell culture and a simultaneous decrease in aggrecanase concentration mimicking an opposite trend observed in OA progression (Figure 7e,f). The quantitative data collected from ELISA corroborated the increased particle uptake of the acan‐HfONS and decreased uptake of acanase‐PrONS as observed with fluorescence imaging. (Figure S11, Supporting Information)

**Figure 8:**
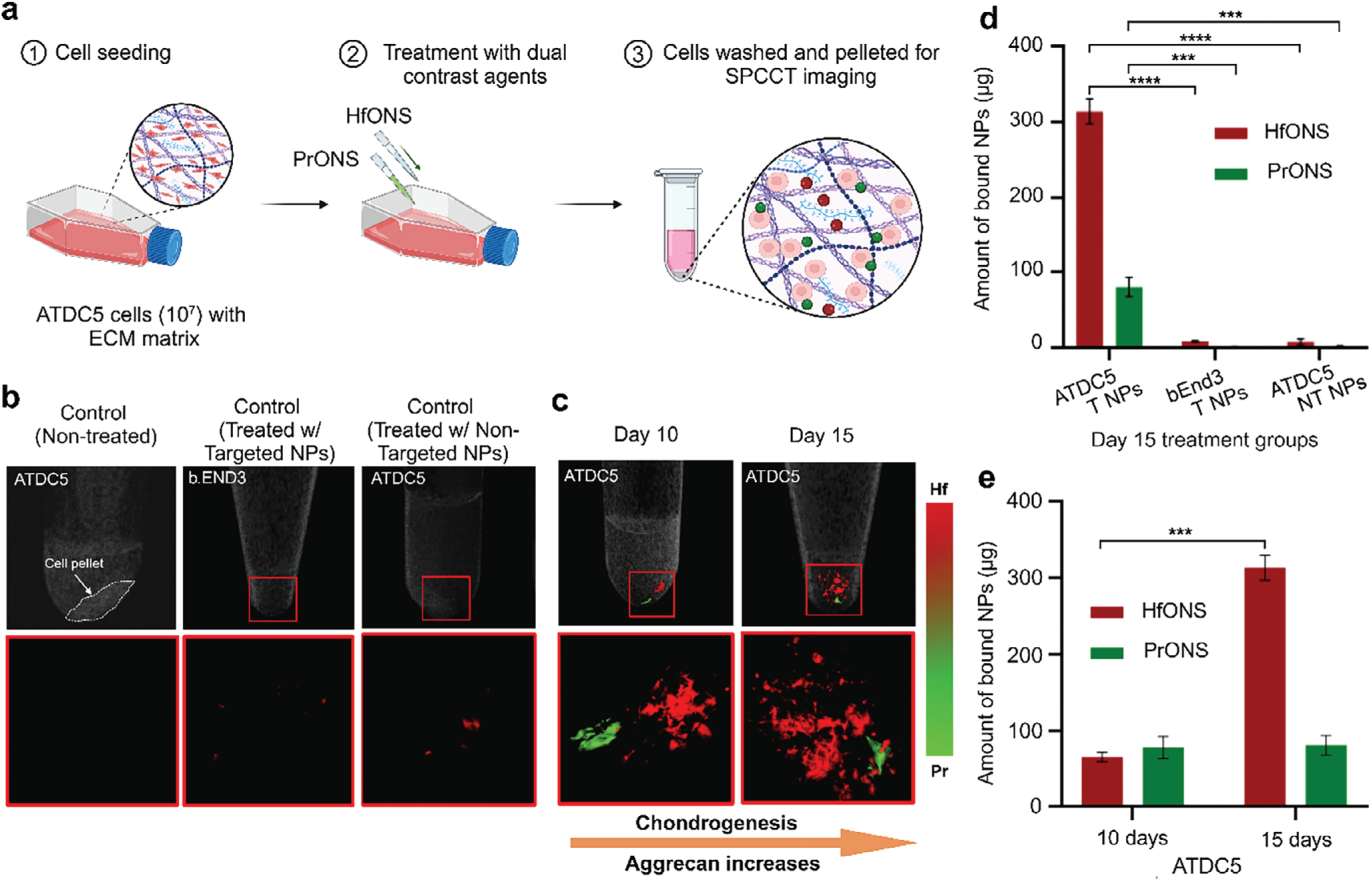
a) Schematic representation of dual nanoprobe treatment in ATDC5 cell culture with multilayer ECM growth; MD applied SPCCT 3D rendering of b) control ATDC5 cell pellet with no NP treatment and control bEnd3 cells (day 7) treated with both Hf/ Pr NPs and control ATDC5 cells (day 15) treated with non‐targeted NPs; c) treated ATDC5 cells at day 10 and day 15 of multilayer ECM growth showing aggrecan production with chondrogenesis; zoomed in images (below) showing signals obtained from selective binding of acan‐HfONS and acanase‐PrONS nanoprobes in cell pellets; Bar plots showing quantification of bound nanoparticles in µg measured by MIQ algorithm in d) ATDC5 treated with targeted nanoparticles (T NPs), bEnd3 treated with targeted nanoparticles (T NPs) and ATDC5 treated with non‐targeted nanoparticles (NT NPs) cell pellets; e) ATDC5 cell pellets at day 10 and day 15 treated with T NPs; color channel: Hf – red, Pr – green, data presented as mean ± standard deviation (*N* = 3). The one‐way ANOVA showed that the nanoparticle uptake was significantly different between ATDC5 treated with targeted and non‐targeted Hf/Pr particles as well as between ATDC5 and bEND3 cells treated with targeted Hf/Pr particles. Significant difference was also observed in targeted Hf NP uptake between ATDC5 day 10 and day 15 treatment groups. ***p‐value <0.001, ****p‐value <0.0001.

**Figure 9:**
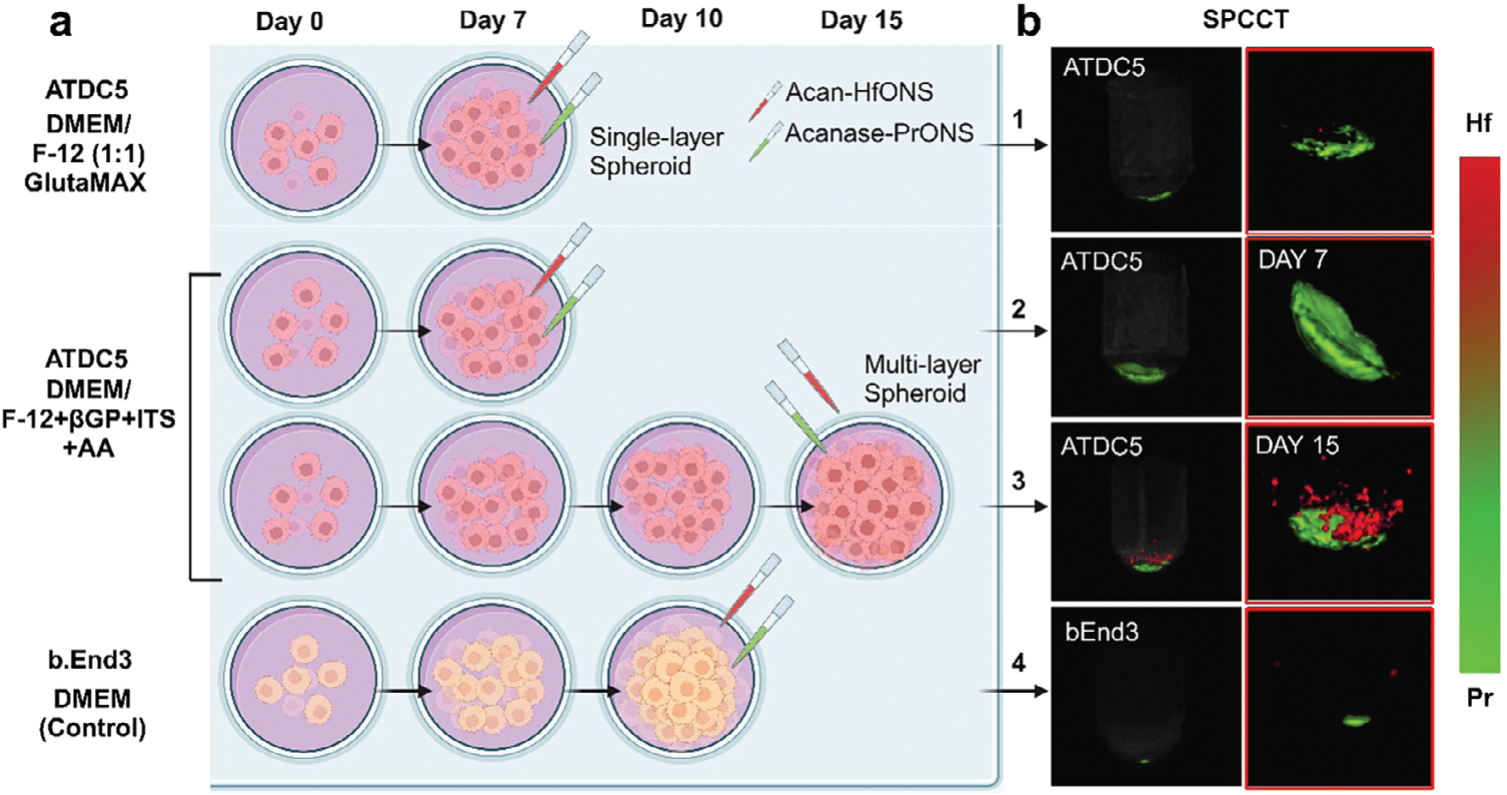
a) Schematic showing spheroid cell culture treatment groups; 1‐ ATDC5 single layer spheroids cultured with DMEM/F‐12 Glutamax and treated with nanoprobes at day 7; 2‐ ATDC5 spheroid culture supplemented with βGP, ITS and AA for ECM production and treated at day 7; 3‐ ATDC5 multi‐layer spheroid culture supplemented with βGP, ITS and AA for ECM production and treated at day 15; 4‐ bEnd3 control spheroids treated at day 10; b) MD applied SPCCT 3D rendering showing selective uptake of the targeted nanoprobes in ATDC5 spheroids with increased Hf signal at day 15 due to excess ECM production and negligible uptake in bEnd3 control spheroids, color channels: Hf – red, Pr – green.

In monolayer culture, ATDC5 cells adhere which allows easy observation and manipulation of the cells, as well as straightforward analysis of aggrecan and aggrecanase protein expression. We performed a quantitative analysis of the binding specificity of the nanoprobes using SPCCT. Treated cell pellets at day 15 of ECM development showed ≈3 times higher Hf signal due to increased aggrecan production compared to day 10 cell pellet with a slight decrease in Pr signal as expected. Brain endothelial (bEnd3) cells were used as a control non‐chondrogenic cell line that does not produce the targeted proteins aggrecan and aggrecanase. bEnd3 cells showed negligible non‐specific uptake of the nanoprobes, which validates the highly selective binding of the targeted particles. (**Figure** **8**) Although monolayer cultures are typically used to mimic early stages of chondrogenesis, such as studying cell proliferation, differentiation, and response to growth factors, the spheroid culture of ATDC5 cells aggregate into a 3D cluster format that more closely mimics the in vivo environment of cartilage tissue, as chondrocytes naturally exist in a 3D matrix.^[^ ^45^ ^]^ Spheroid cultures allow for the investigation of cell–cell interactions, ECM production, and tissue‐level responses to mechanical stimuli.^[^ ^46^ ^]^ Higher ECM construction expresses higher aggrecan levels and these spheroids are often used to study later stages of chondrogenesis, such as cartilage maturation, hypertrophy, and mineralization.^[^ ^47^ ^]^ In multilayer spheroid culture, multiple layers of individual ATDC5 spheroids are stacked on top of each other and provide an even more complex and physiologically relevant model of native articular cartilage tissue that can mimic OA.^[^ ^47^ ^]^ With time, the expression of aggrecan is expected to increase in the ECM of multilayer spheroids with a slight decrease in aggrecanase concentration in the chondrocytes. To better understand the interaction and binding specificity of the nanoprobes, this format was developed using Nunclon sphera well plates. The spheroids were developed at different time points, and treated with the nanoprobes, and the particle uptake in the pellets was visualized using SPCCT. We observed a higher amount of Hf signal in the 15‐day multilayer spheroid culture as acan‐HfONS accumulated more in the ECM construction compared to day 7. Similarly, decreasing aggrecanase concentration was observed as the Pr signal decreased with time due to reduced acanase‐PrONS production compared to the initial 7 days in the spheroid cell pellet. This shows the selective binding of both nanoparticles in the spheroid model due to varying concentrations of protein production. A single‐layer spheroid was also developed and compared to multilayer spheroids by altering the culture media and growth supplement which showed smaller spheroids with reduced particle uptake as expected. bEnd3 spheroid cells were developed as a control group to test the non‐specific uptake of the nanoprobes and scanned using SPCCT. Minimal Hf and Pr signals were observed as these cells do not express OA markers and the non‐specific accumulation of targeted particles to aggrecan and aggrecanase are negligible. (**Figure** **9**)

### 2.5 In Situ Detection and Delineation of Hf and Pr Nanoprobes in a Biological Environment using SPCCT

**Figure 10:**
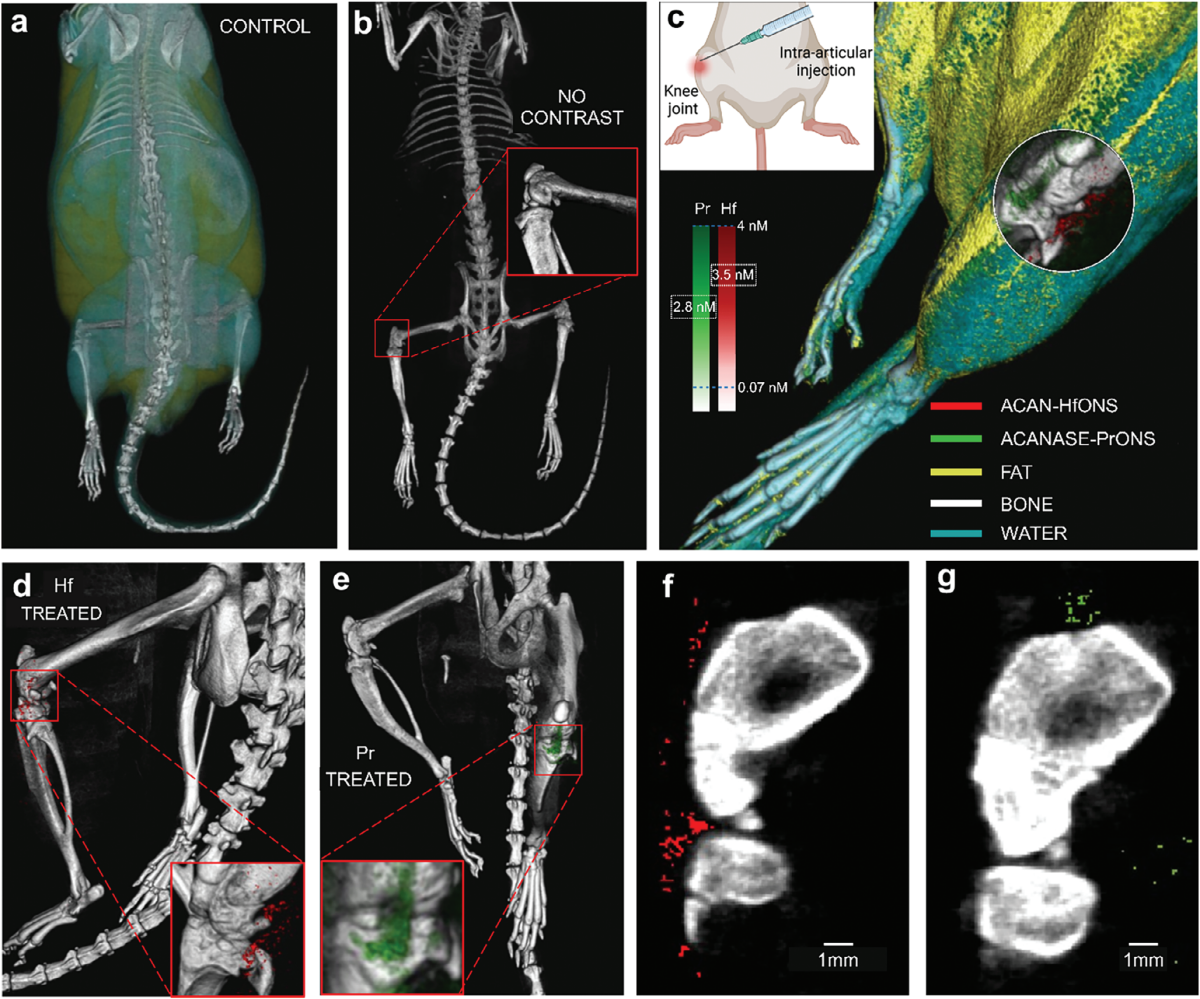
SPCCT imaging of dual‐targeted contrast agents in a treated rat knee joint a) Control rat scan showing calcium, lipid, and water channels; b) Calcium channel delineated from the control rat scan; inset shows magnified view of the untreated knee joint with no nanoparticle contrast; c) Material decomposition of treated rat scan showing Pr (green) and Hf (red) signals at the left knee joint; inset shows a schematic representation of intra‐articular injection of contrast agent into the rat knee, Both targeted Hf/Pr nanoparticles were injected at a concentration of 8 mg mL^−1^, scale bar shows the color intensity of estimated nanoparticle concentrations (nM) detected by SPCCT for both Hf and Pr in the treated rat knee joint; Individual channels visualized in the treated rat d) Red channel showing HfONS signal; e) green channel showing PrONS signal in the knee joint; Axial slice view of f) HfONS; g) PrONS signals (scale bar = 1 mm).

We further explored the feasibility of specifically identifying the developed contrast agents in situ in a biological environment using MARS SPCCT by injecting the nanoparticles into healthy knee joints of Long‐Evans rats (250 g, 7 months old). The animals were split into two groups, control and treated rats to differentiate the distinct nanoparticle signals and to account for background noise. To begin with, control scans were done without nanoparticle treatment and reconstructed using the MARS scanner and the developed Hf/Pr scanning protocol as a baseline measurement. Precise intra‐articular injections^[^ ^48^ ^]^ of both contrast agents were made into the left knee joint space of the rats using sterile 25G needle syringes at concentrations based on previously reported methods.^[^ ^49^ ^]^ Post injection of the nanoparticles, a 1‐h incubation time was allowed for the particles to bind. This was followed by an additional cleaning procedure by administering 5 µL regular saline at the joint to remove any unbound particles. The treated rats were scanned immediately using the same protocol and reconstruction parameters as the control group. The scan time for each rat was ≈40 min. Material decomposition was applied and the scans were visualized using MARSVision to delineate the Pr and Hf channels. **Figure** **10**a,b shows the 3D reconstructed SPCCT images of the control animal showing the lipid, water, and calcium channels with no contrast identified from the Pr and Hf channels. Figure 10d–g shows the individual Hf and Pr signals from the left knee joints of the treated animals. These results demonstrate the possibility of detecting this metal nano pair in a biological environment using the established SPCCT protocol as well as differentiating them from bone and soft tissue with high specificity and minimal overlap. (Figure 10c)

Although the imaging shown here demonstrates that individual signals from the nanoparticles can be clearly detected using SPCCT, it is important to note that there may be variations in perfusion, clearance rates, and systemic tracer kinetics that make it difficult to extrapolate these results to physiological conditions. Within the given experimental configuration, the available data serves as a proof‐of‐concept for the technology's signal detection and delineation capabilities in situ. Furthermore, the use of praseodymium‐based nanoparticles for imaging is new. There is no known literature on SPCCT contrast or in vivo toxicity, in contrast to hafnium‐based nanoparticles that have been previously studied as contrast agents for SPCCT and have low in vivo toxicity documented in our prior work.^[^ ^50^, ^51^ ^]^ Initial in vitro cytotoxicity evaluations conducted on chondrogenic cell lines reveal negligible to no toxicity, which may be explained by the CS capping on the surface of the nanoparticles. To assess these nanoparticles' safety profile and viability for clinical translation, more research is necessary to investigate their long‐term effects in vivo, including their biodistribution, clearance, and potential accumulation in organs. These nanoparticle platforms are currently being investigated as in vivo contrast agents to test their feasibility, biocompatibility, accumulation, and biodegradability, aiming at future clinical translational studies using an OA animal model.

## 3 Conclusion

Multicontrast k‐edge based imaging using SPCCT offers huge potential for molecular imaging of diseases. Without adequate early diagnosis and longitudinal tracking, diseases such as OA can progress unnoticed until clinical presentation, when they reach an advanced and irreversible stage. At an advanced stage, exacerbation of symptoms leads to poor lifestyle and disability, which increases the financial burden on the healthcare system. This is all because of missed opportunities for early intervention due to the lack of tools that enable early diagnosis and longitudinal disease tracking. Enhancing imaging techniques to detect early cartilage changes at a molecular level will improve diagnosis and monitoring. The study, detailed herein, aims to address this issue by developing SPCCT contrast nanoprobes from hafnium and praseodymium that can molecularly target complementary biomarkers of interest and enable early diagnosis and longitudinal monitoring of disease progression. These nanoprobes can selectively bind to antigens and can be quantified accurately using the SPCCT MIQ algorithm, which could significantly improve disease management leading to improved lifestyle and reduced disability among affected patients. Ultimately, this will reduce the associated financial cost in the healthcare system. The study demonstrates the capability of the developed k‐edge nanoprobes and SPCCT platform in detecting low nanomolar concentrations of bound nanoparticles and shows their potential in detecting critical stages of the disease before symptom development and clinical presentation with advanced stages. The study also shows the potential of the SPCCT platform to be able to detect accurate signals from low concentrations of the nanoprobes in an in situ environment. Future work will test the efficiency of these nanoprobes as in vivo contrast agents using an OA animal model.

## 4 Experimental Section

### Materials

All solvents were of reagent grade and purchased from Sigma–Aldrich Inc. (Saint Louis, MO, USA). Praseodymium (III) nitrate hexahydrate salt, CS sodium salt from bovine cartilage (average M_n_: 10–40 kDa, Lot # SLCF6270), and ammonium hydroxide (28‐30%) reagent were obtained from Sigma–Aldrich Inc. Hafnium (IV) nitrate salt was purchased from Onyxmet. ATDC5 mouse teratocarcinoma cell line was purchased from Sigma–Aldrich. The aggrecan (PG) Mouse aggrecan ELISA kit was purchased from Bioss USA and the mouse ADAMTS5 ELISA kit was from MyBiosource. The Pierce FITC Antibody Labeling Kit was purchased from Thermofisher Scientific and the EZLabel Antibody Cy5 labeling kit from Biovision. DMEM/F‐12 cell culture media, GlutaMAX supplement, Insulin‐Transferrin‐Selenium (ITS ‐G) (100X), and Penicillin‐Streptomycin (5000 U mL^−1^) were purchased from Thermofisher Scientific. ADAMTS5 polyclonal antibody (bs‐3573R) was purchased from Bioss antibodies and aggrecan monoclonal antibody (BC‐3) (MA3‐16888) was purchased from Thermofisher Scientific.

### Methods—Synthesis of Chondroitin Sulfate Capped Nanoparticles

The synthesis of PrONS was based on the nitrate reduction of Pr containing precursor praseodymium (III) nitrate hexahydrate (Pr (NO_3_)_3_.6H_2_O) salt. 1 mL of 1 m praseodymium nitrate hexahydrate was dissolved in 2 mL of 0.4 mmol of CS solution in DI water. The precursor was added dropwise to a 25 mL round bottom flask containing 6 mL of concentrated ammonium hydroxide solution (28–30%) while stirring. The reaction mixture was heated to 90 °C for 1 h under a reflux condenser. After 1 h of heating, the reaction mixture was allowed to cool to room temperature and stirred overnight. The particle suspension was then spun at 7100 rcf for 10 min to settle large aggregates. The supernatant was removed, and the settled particles were washed 3 times with DI water to remove unreacted Pr nitrate, ammonium hydroxide, and CS. The particles were either dried overnight under vacuum or lyophilized for further use. A similar synthesis process was used for synthesizing HfONS capped with CS using reduction of hafnium (IV) nitrate (Hf (NO_3_)_4_) salt.

### Ligand Functionalization of Nanoparticles

PrONS and HfONS were conjugated respectively to ADAMTS5 polyclonal antibody (1 µg mL^−1^ stock) targeting aggrecanase and aggrecan monoclonal antibody (1 mg mL^−1^ stock) targeting aggrecan glycosylated protein. The conjugation was made via EDC‐NHS coupling for multiplexed detection in both in vitro and ex vivo models. A mixture of 40 mg mL^−1^ stock solution of each particle with 0.06 mmol EDC, 0.05 mmol DMAP, and 25 µL of 1:200 dilution of respective antibody solution (stock concentration) was stirred in a 25 mL RB flask for 48 h at room temperature. The mixtures were centrifuged and washed to remove excess unbound antibodies and resuspended in water and stored at 4 °C for further use in cell studies.

### Measurement of Hydrodynamic Diameter using Dynamic Light Scattering (DLS)

The average hydrodynamic diameter of the PrONS and HfONS were measured using a Malvern Nano‐S Zetasizer. The particles were diluted to an optimum extent in disposable cuvettes before each measurement and multiplied by the dilution factor to obtain the results.

### Transmission Electron Microscopy (TEM)

A high amplitude ultra‐probe sonicator (Q700, Qsonica Sonicators, Newtown, CT) was used to resuspend the particles in water in order to obtain a homogenous well‐dispersed suspension. 5 µL of this suspension was drop cast onto carbon‐coated copper grids (400 mesh size). After 3 mins, the excess liquid was wicked out using filter paper and the grid was air dried before imaging using the Hitachi HT7800 120 kV TEM with an AMT Nanosprint15 B digital camera at the UMBC campus. The anhydrous diameters of both PrONS and HfONS were calculated using ImageJ software. The FEI Talos F200X at the MCL PSU campus was used to image these particles using STEM modes for elemental analysis using EDS/HAADF.

### Fourier Transform Infrared Spectroscopy (FT‐IR)

The particle samples in powder form were placed directly onto the diamond crystal and the transmissions were measured under the attenuated total reflectance (ATR) sampling technique using the Agilent Cary 630 FTIR.

### X‐Ray Diffraction Measurements (XRD)

Powder X‐ray diffraction was used to check the chemical structure of the nanospheres. 40 µL of water‐dispersed samples were drop cast onto silicon zero background wafers and dried overnight under vacuum. The diffraction data was collected from 5 to 70° 2θ using a Malvern Panalytical Empyµrean instrument fitted with a copper (Kα1 = 1.540598 Kα2 1.544426 Å) long‐fine‐focus X‐ray tube operated at 45 kV and 40 mA.

### X‐Ray Photoelectron Spectroscopy

The samples were prepared by drop casting 40 µL of water‐dispersed samples onto silicon wafers and dried overnight under vacuum. XPS experiments were performed using a Physical Electronics VersaProbe III instrument equipped with a monochromatic Al kα x‐ray source (hν = 1486.6 eV) and a concentric hemispherical analyzer.

### Raman Spectroscopy

Samples were prepared by drop casting water‐dispersed particle solutions before and after surface functionalization with respective antibodies onto clean silicon substrates. Samples were dried under vacuum at RT and the spectrum was measured using the Renishaw InVia confocal Raman microscope at a laser excitation wavelength of 785 nm (power 1%, grating 1200 l nm^−1^). A 50X objective lens was used for data collection with Raman shift ranging, from 100 to 1800 cm^−1^. All spectra were obtained by averaging at least 10 individual spectra obtained with an exposure time of 10 s. Renishaw 51P859 detector was used for these experiments.

### Zeta Potential

The zeta potentials of the particles were measured in triplicates using the Malvern Zetasizer Nano Series by injecting optimally diluted concentrations of the water‐dispersed nanoparticle solutions.

### ATDC5 Cell Culture

Chondrogenic ATDC5 cells were cultured in a differentiation medium [DMEM/F‐12 (1:1) with GlutaMAX I containing 5% FBS, 1% sodium pyruvate, and 0.5% Pen Strep] in a T‐75 flask. The cells were incubated in a humidified atmosphere (37 °C, 5% CO_2_) till they reached confluency. The cells were passaged using 0.25% Trypsin‐EDTA and the medium was renewed every 2 to 3 days. For ELISA and imaging experiments, the subcultures were further cultured by adding 1% insulin transferrin selenium to the differentiation medium at a density of 70000 cells cm^−2^ in a 24‐well plate. The cells were incubated for 6 days till they reached confluency and the medium was supplemented with 10 mM β‐Glycerophosphate disodium salt hydrate (βGP) and 50 µg mL^−1^ L‐ascorbate‐2‐phosphate (ascorbic acid). Cells were further incubated in a humidified atmosphere (37 °C, 5% CO_2_) for up to 25 days, and the medium was changed every 2 to 3 days. Similarly, subculture cells were plated in larger T‐75 plates in multiple batches to observe the aggrecan GAG levels and chondrogenesis at 10‐ and 15‐day timepoints using SPCCT. 3D spheroid cultures of ATDC5 were maintained in faCellitate Biofloat 96‐well plates with 6000 cells per well. Spheroids were cultured in DMEM/F‐12 (1:1) with GlutaMAX I containing 5% FBS, 1% sodium pyruvate, and 0.5% Pen Strep. For multi‐layer spheroid cultures, 2000 cells per well were cultured by adding 1% insulin transferrin and selenium to the differentiation medium, and after 4 days of confluency, the medium was supplemented with 10 mM βGP and 50 µg mL^−1^ L‐ascorbate‐2‐phosphate. Cells were further incubated in a humidified atmosphere (37 °C, 5% CO_2_) for up to 10–15 days, and the medium was changed every 2 to 3 days. Aggrecan and aggrecanase levels in chondrogenesis were observed at different time points for these spheroid models. Human bEnd3 cells were also cultured as control 3D spheroids in Dulbecco's Modified Eagle's Medium with 10% FBS and 0.5% Pen Strep.

### Confocal Microscopy

Live cell brightfield and fluorescence images were procured using the Zeiss monochromatic microscope, Hitech Instruments at 20X objective to check cell viability and particle uptake using the FITC and Cy5 fluorescence channels. ImageJ software was used for semi‐quantitative visualization of the fluorescence channels.

### MTT Assay

The assay was performed in 96‐well flat bottom microplates (Corning, NY, USA). Three independent experiments were performed for each particle in the concentration range of 0.01, 0.03, 0.0625, 0.125, 0.25, 0.5, and 1 mg mL^−1^. The total number of wells included a first batch of cells treated with acanase‐PrONS, a second batch treated with acan‐HfONS, and a third batch of wells treated with both particles simultaneously including control cells for each experiment. 20000 cells in 200 µl of cell culture media were added to each well and incubated in a CO_2_ incubator for 24 hrs. After 24 hrs, the predetermined concentration of targeted nanoprobes was added to each well and incubated for 6 hrs. After 6 hrs, the cell culture supernatant was replaced with fresh media and, and the cells were incubated for 42 hrs. After 42 hrs, 20 µL of 5 mg mL^−1^ stock solution of MTT reagent was added to each well and incubated for 3 to 4 hrs. At 4 hrs, DMSO was added to each well to form the formazan crystals, and the UV absorbance was recorded using a plate reader at 570 nm to check cell viability.

### Ex Vivo Model

Human phalanges that were ethically and legally obtained were purchased from Skulls Unlimited Inc. (Carlsbad, CA). The ligand‐coated acanase‐PrONS and acan‐HfONS stocks (40 mg mL^−1^) were adequately mixed with the respective antigens. 1.5 mL of the ADAMTS5 antibody targeted PrONS was complexed with 25 µL of ADAMTS5 protein (0.17 mg mL^−1^ stock) and 1.5 mL of the aggrecan antibody targeted HfONS was complexed with 5 µL of aggrecan protein (2 mg mL^−1^ stock). The mixtures were stirred at room temperature for 30 mins and centrifuged at 7100 rcf for 10 mins. The supernatant was pipetted carefully to remove any unbound antibodies or proteins and the particles were resuspended in water and stored at 4 °C until further use. Varying predetermined ratiometric volumes (0, 50, 100, 150, and 200 µL) of antigen‐bound targeted nanoprobes were injected into the human phalangeal joint cartilage (5% agarose gel) to mimic increasing OA severity from a healthy joint to a severe OA joint.

### Animal Studies

To investigate the potential of detecting low concentrations of both Hf/Pr contrast agents using SPCCT in an in vivo environment, normal Long‐Evans rats were treated by intra‐articularly injecting the nanoparticles in their knee joints and the animals were scanned using the MARS scanner. The experimental protocol received ethical approval and was approved by the Institutional Animal Care and Use Committee (IACUC), Pennsylvania State University, and was consistent with local, state, and federal regulations as applicable for the humane use of laboratory animals. The animal experiments complied with the relevant ethical regulations for animal testing and research and were carried out in accordance with the approved guidelines.

### Inductively Coupled Plasma–Optical Emission Spectroscopy (ICP‐OES)

Pr and Hf metal concentrations of the particles were measured using the Agilent 5800 ICP‐OES spectrometer. The samples were fully digested in aqua regia, and the final solutions were prepared as a 35x diluted sample in 2% nitric acid to measure the metal concentrations. Separate ICP standards for praseodymium and hafnium (*Trace*CERT, Sigma–Aldrich) were used to create calibration curves using the inbuilt ICP Expert software.

### Nanoparticle Quantification

Absolute Hf/Pr concentrations were derived from the MARS material decomposed (MD) images with the concentrations extrapolated using the ICP standard curve and a numerical calculation was done with an assumption of 15 nm sized Hf particles comprising ≈4.2 × 10^11^ number of nanoparticles in 50 µL injected dose and 21 nm sized Pr particles comprising ≈3.5 × 10^13^ number of nanoparticles in 50 µL injected dose. The nanoparticle quantifications were made by first calculating the total number of nanoparticles with the assumption that the particles were spherical and occupied unit volume equivalent to 4/3π*r* ^3^. This number was used to calculate moles of nanoparticles (hafnium/praseodymium) per mL by applying Avogadro's number and represented in nanomolar concentrations.

### Statistical Analysis

Statistical data were analyzed via GraphPad Prism 9.5.1. All plots were presented as mean ± standard deviation (SD) obtained after repeated measures. Statistical analysis was performed using a one‐way repeated measure analysis of variance (ANOVA) to evaluate assay response toward the various sample groups under investigation. Differences between experimental groups and control groups were considered statistically significant with a p‐value < 0.0001. The sample size applied for each experiment was indicated in the figure captions.

## Conflict of Interest

Prof. Pan is the founder/co‐founder of five university based start ups. None of these entities, however, supported this work.

## Supporting information



Supporting Information

## Data Availability

The data that support the findings of this study are available in the supplementary material of this article.
