# Supplementary material for: Targeted K‐Edge Nanoprobes From Praseodymium and Hafnium for Ratiometric Tracking of Dual Biomarkers using Spectral Photon Counting CT
Source: Adv Sci (Weinh). 2024 Oct 7;11(46):2408408. doi: 10.1002/advs.202408408 (PMC11633496; doi:10.1002/advs.202408408)
Supplement: Supplementary file 1 — Supporting Information [file ADVS-11-2408408-s001.docx]

Supporting Information

©Wiley-VCH 2023

69451 Weinheim, Germany

Targeted K-edge Nanoprobes from Praseodymium and Hafnium for Ratiometric Tracking of Dual Biomarkers using Spectral Photon Counting CT

*Nivetha Gunaseelan,^1,2,3^ Parikshit Moitra,^1,2^ Pranay Saha,^1^ Teresa Aditya,^1^ Mahdieh Moghiseh,^4^ Kevin Jonker, ^4,5^ Steven Gieseg,^4,5,6^ Anthony Butler,^4,6^ Fadia Kamal,^7^ and Dipanjan Pan^*1,2,3^*

1 Huck Institutes of the Life Sciences, Department(s) of Biomedical Engineering, Nuclear Engineering, Materials Science and Engineering, The Pennsylvania State University, 101 Huck Life Sciences Building, University Park PA 16802 USA

2 Department of Pediatrics, Centre for Blood Oxygen Transport & Hemostasis, University of Maryland Baltimore School of Medicine, Baltimore, Maryland 21201, USA

3 Department of Chemical & Biochemical Engineering, University of Maryland Baltimore County, Baltimore, Maryland 21250, USA

4 MARS Bioimaging Limited, 68 Saint Asaph Street, Christchurch Central City, Christchurch, 8011, New Zealand

5 School of Biological Sciences, University of Canterbury, Private Bag 4800, Christchurch, 8041, New Zealand

6 Department of Pathology and Biomedical Science, University of Otago, 2 Riccarton Avenue Christchurch, 8011, New Zealand

7 Center for Orthopaedic Research and Translational Science, Department of Orthopaedics and Rehabilitation, Penn State College of Medicine, The Pennsylvania State University, Hershey, PA 17033, USA

* E-mail: dipanjan@psu.edu

| **Table of Contents** |
| --- |
| Results  Figures  Figure S1…………………………………………………………………………………………S3 |
| Figure S2…………………………………………………………………………………………S4 |
| Figure S3…………………………………………………………………………………………S4 |
| Figure S4…………………………………………………………………………………………S5 |
| Figure S5…………………………………………………………………………………………S6 |
| Figure S6…………………………………………………………………………………………S7 |
| Figure S7…………………………………………………………………………………………S8 |
| Figure S8…………………………………………………………………………………………S9 |
| Figure S9…………………………………………………………………………………………S10 |
| Figure S10...……………………………………………………………………………………...S11 |
| Figure S11………………………………………………………………………………………..S11 |
| Tables  Table S1…………………………………………………………………………………………..S12  Table S2…………………………………………………………………………………………..S12 |
| Table S3…………………………………………………………………………………………..S13 |
| Table S4…………………………………………………………………………………………..S14 |
|  |

**Results**


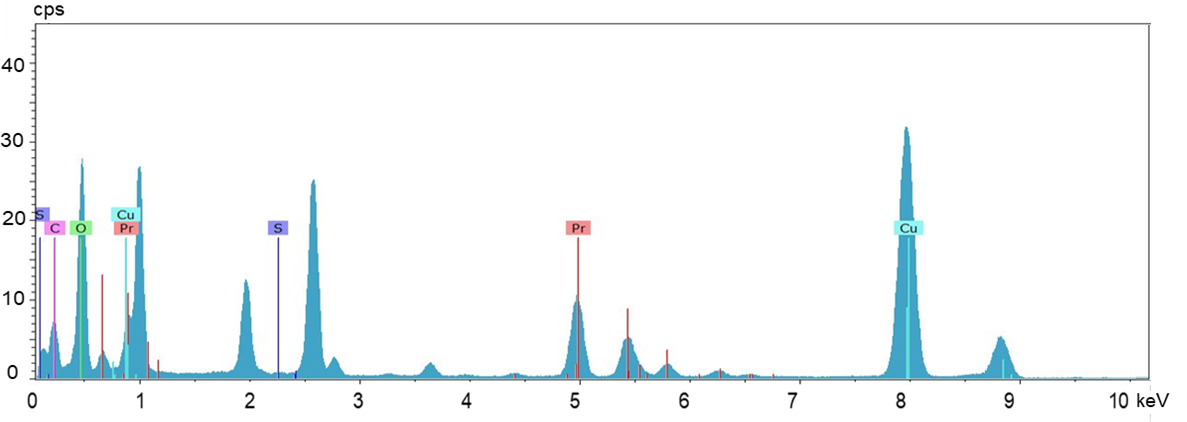


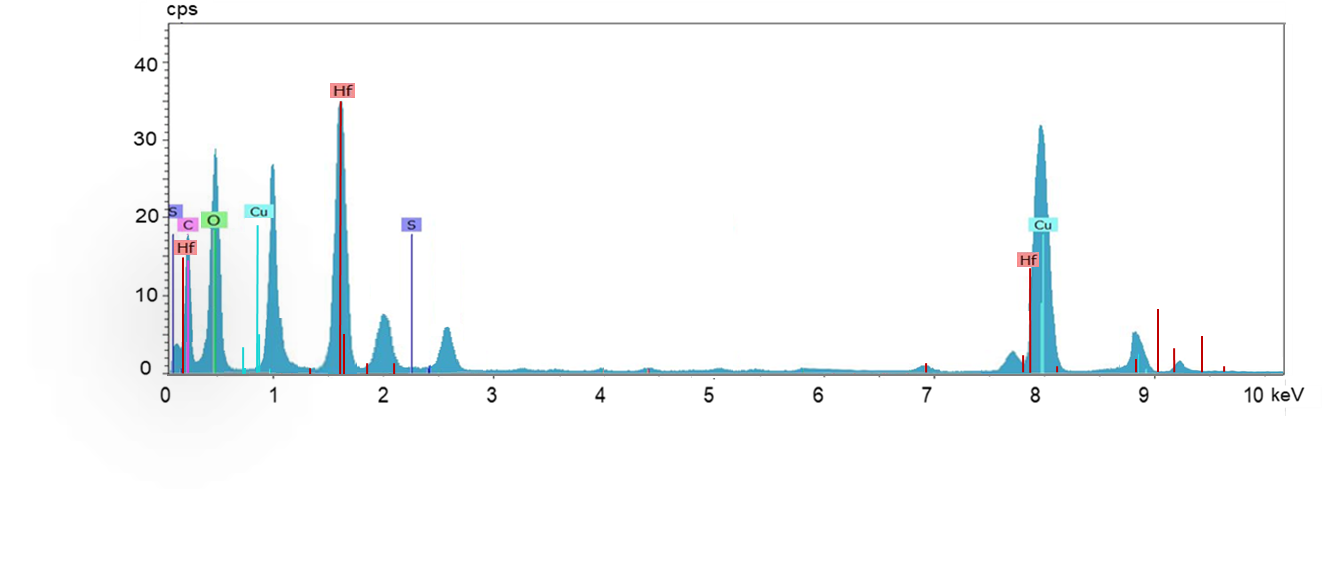


**Figure S1** TEM EDS spectra showing presence of elements Pr, S, O and C in PrONS nanoparticles and Hf, S, O, C in HfONS nanoparticles; the Pr and Hf peaks in the respective spectra show the dominant presence of the metals in the synthesized nanoparticles.


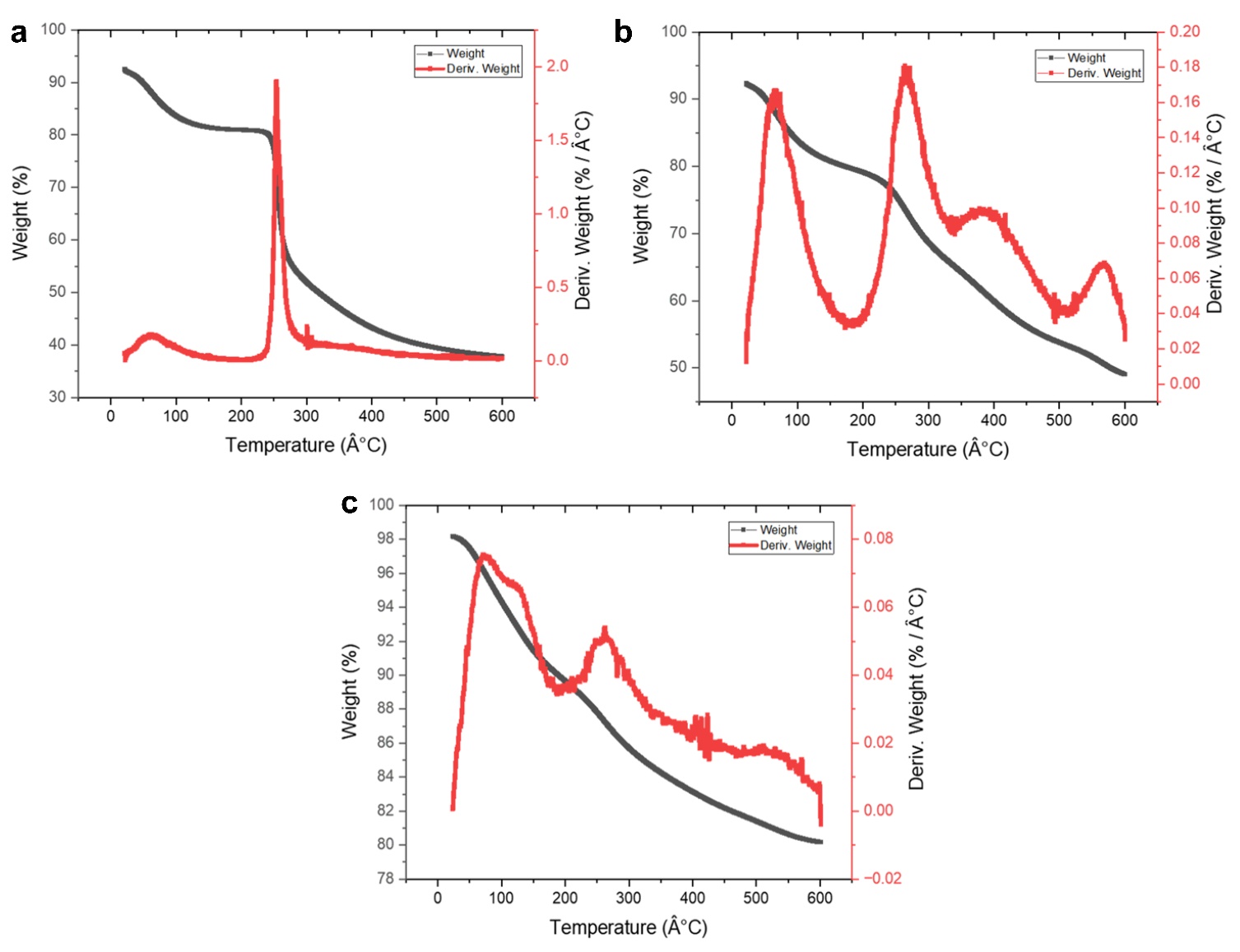


**Figure S2** TGA and the corresponding DTG curves showing decomposition trend for a) CS, b) PrONS and c) HfONS


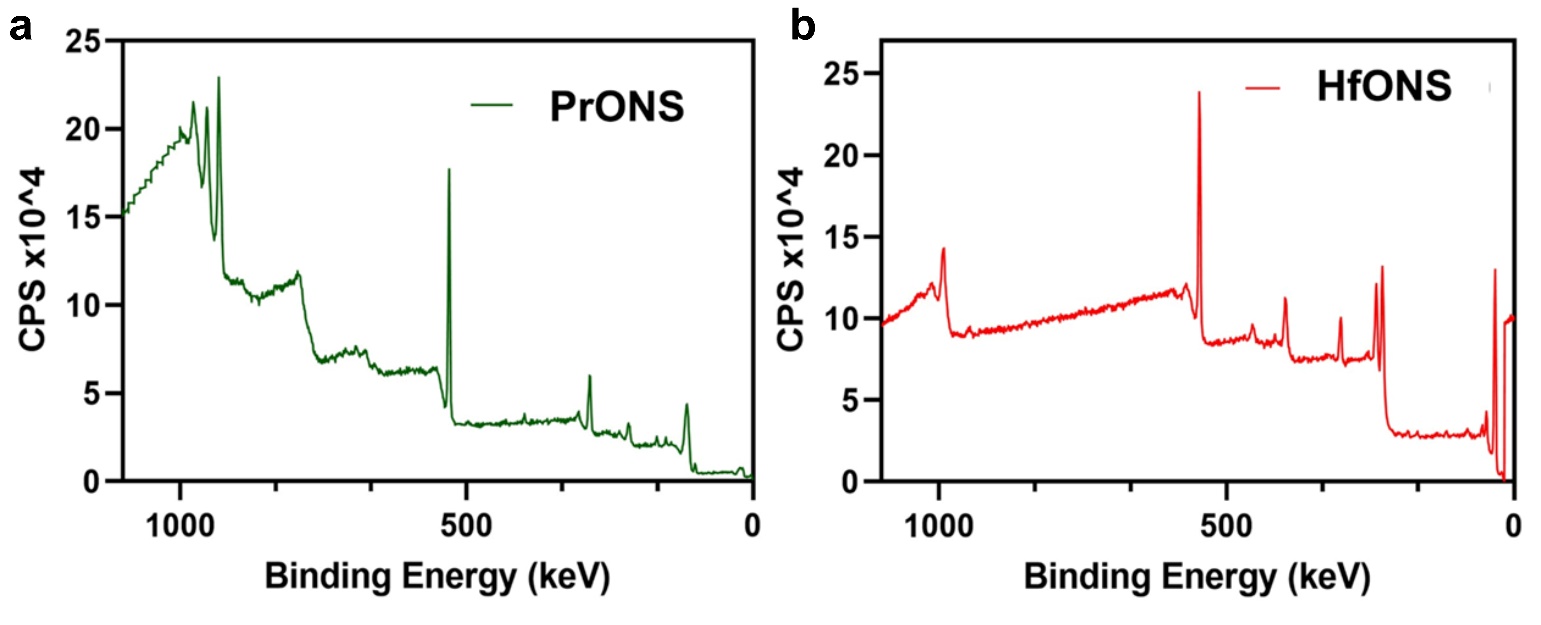


**Figure S3** XPS survey spectra for a) PrONS and b) HfONS


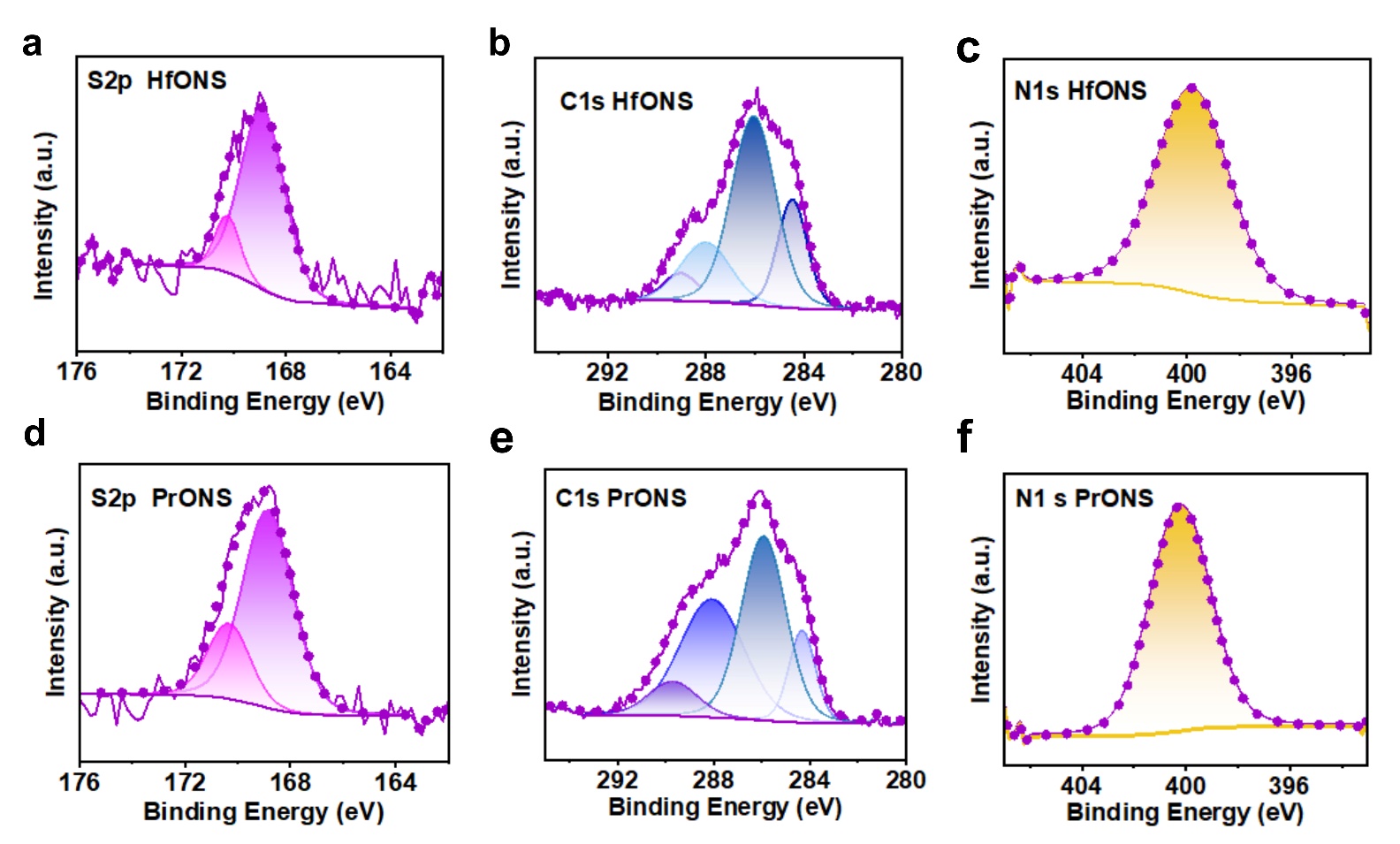


**Figure S4** HfONS XPS spectra revealing a) S2p peaks were observed around 170.23 eV for S2p1/2 and 168.89 eV for S2p3/2, b) three prominent features of C1s peak at 289.03, 288.00 286.04, 284.47 eV that can be assigned to O-C=O, N-C=O/C-OSO_3_, C-O/C-N and C-C/C-H bonds respectively and c) N1s peak at 399.64 eV; XPS spectra for PrONS showing d) S2p peaks of sulfur ascribed to S2p1/2 at 170.33 eV and S2p3/2 at 168.85 eV, e) three prominent features of C1s peak at 289.70, 288.07, 285.92 and 284.32 eV for the above-mentioned carbon groups and f) N1s peak at 399.81 eV


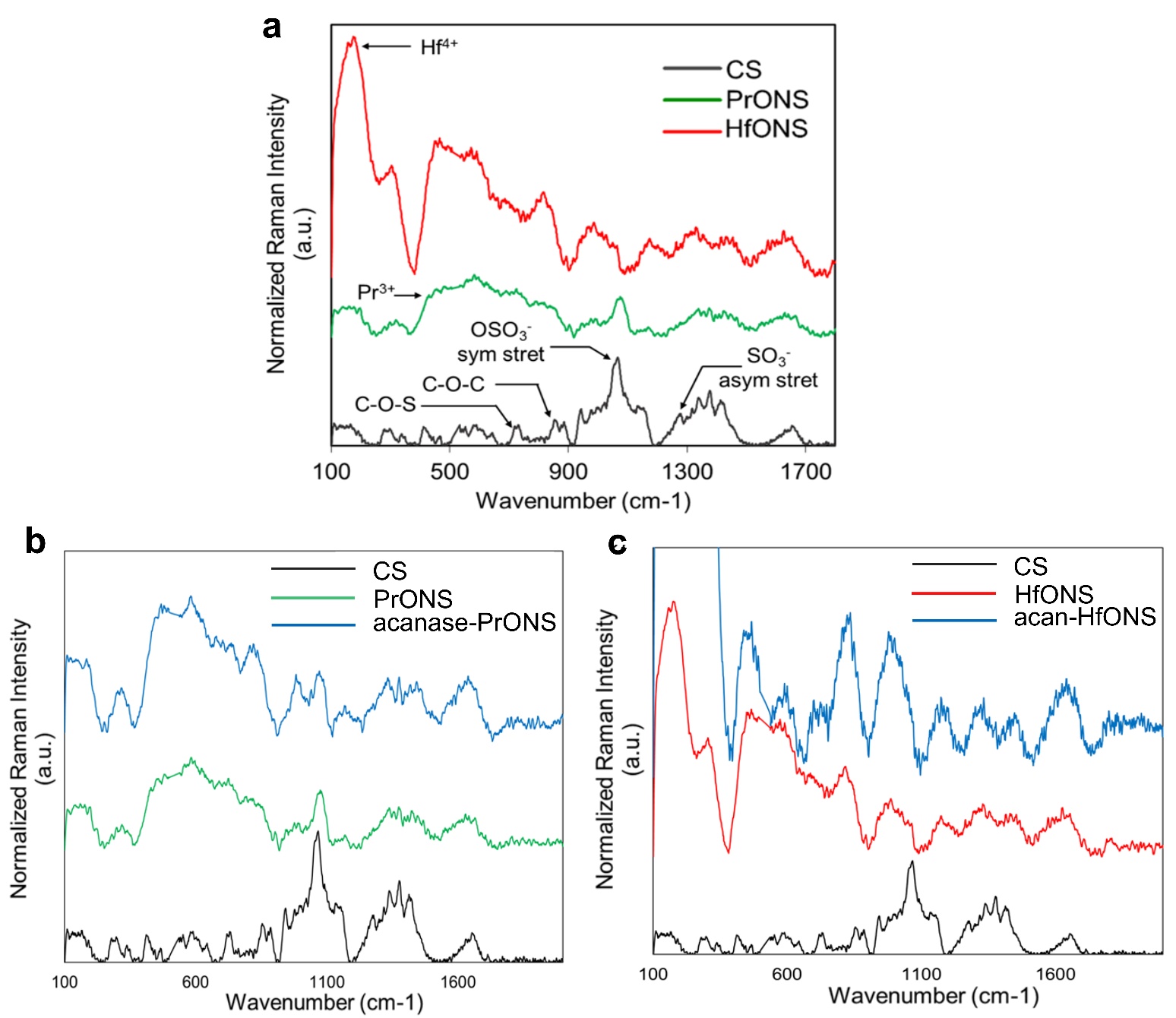


**Figure S5** Raman spectra of a) non-targeted PrONS vs non-targeted HfONS vs CS alone indicating the success of CS capping on the synthesized nanospheres; b) CS, non-targeted and targeted PrONS functionalization of aggrecanase antibody c) CS, non-targeted and targeted HfONS showing functionalization of aggrecan antibody


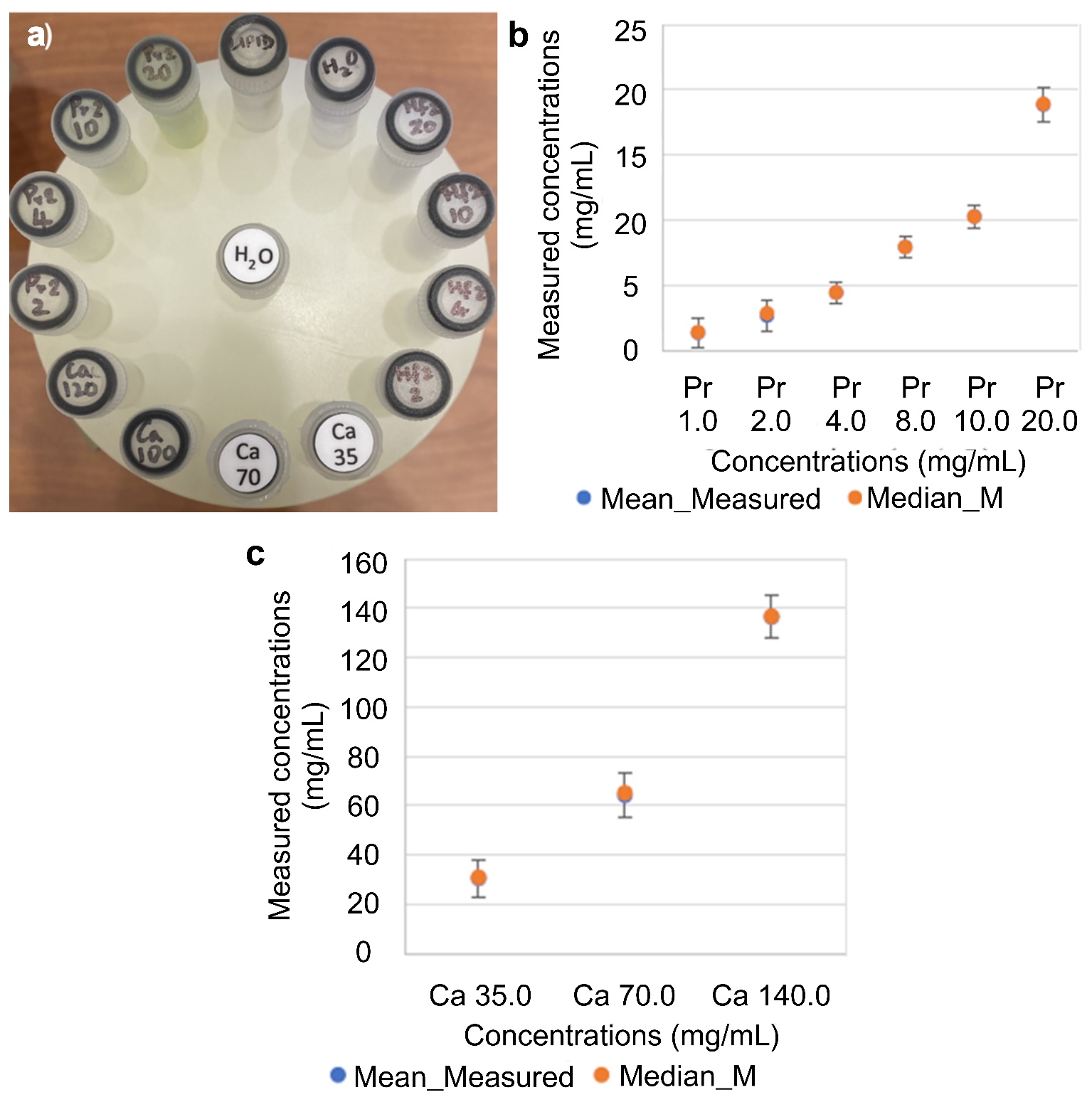


**Figure S6 a)** SPCCT PMMA phantom loaded with PrONS, HfONS and Ca standards, Graphs indicating linear correlation between measured concentration using MIQ algorithm versus actual concentration (mg mL^-1^) for b) Pr dilutions, c) Ca dilutions in the phantom vials. Data is presented as mean *±* standard deviation (*N=3*)


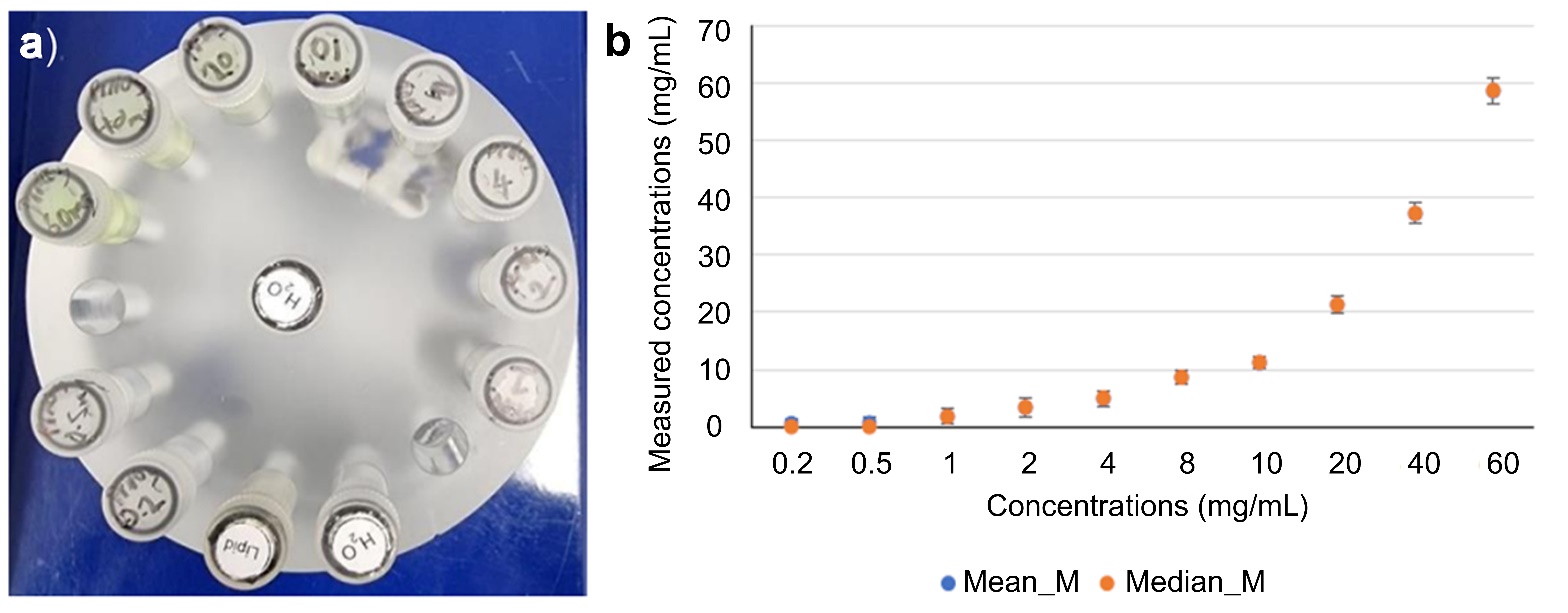


**Figure S7** a) SPCCT PMMA phantom loaded with extended range of PrONS standard solutions (0.2 to 60 mg/mL), lipid and water b) Graph indicating measured concentration of Pr in each vial using MIQ algorithm versus actual concentration of Pr standard dilutions (mg mL^-1^). Data is presented as mean *±* standard deviation (*N=3*)


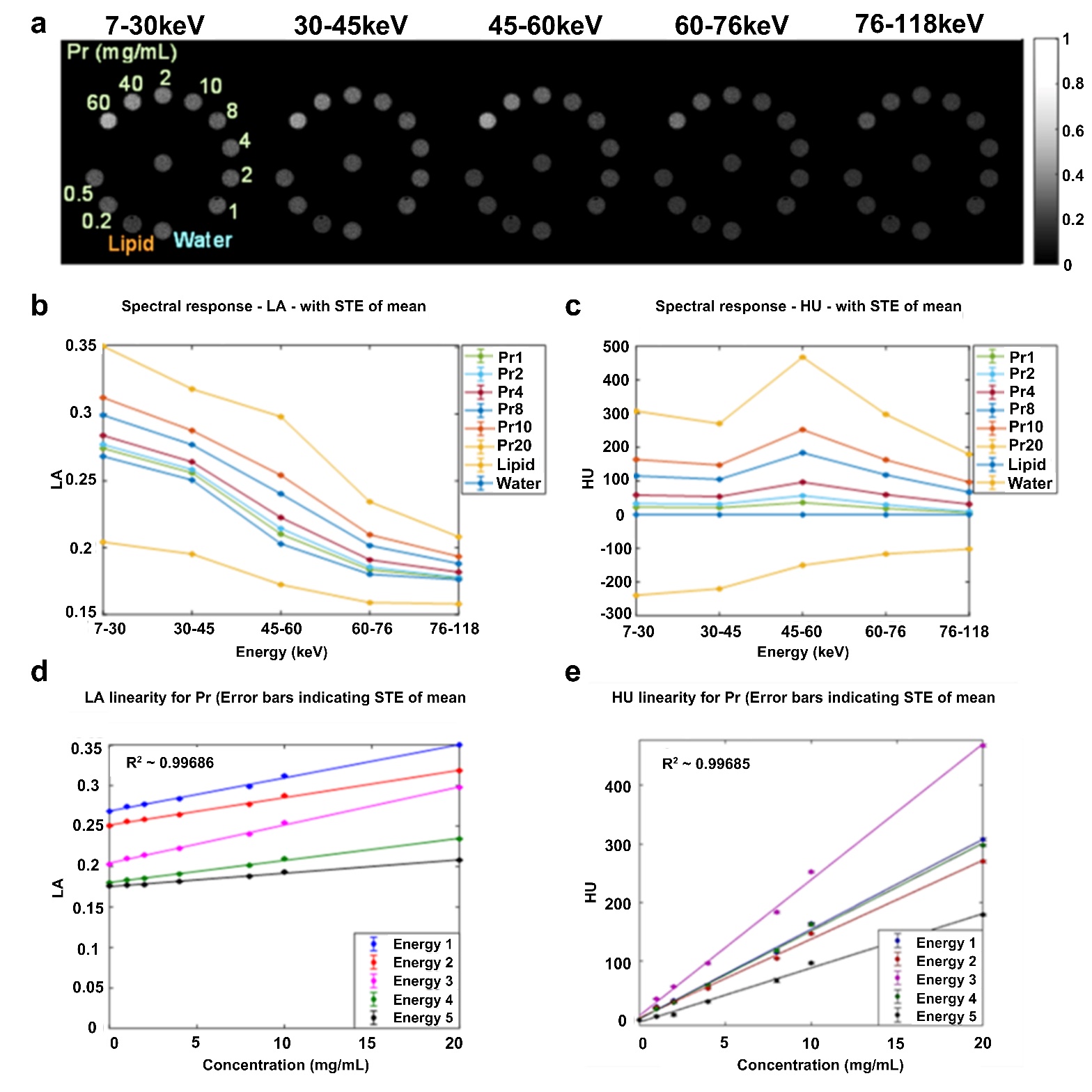


**Figure S8** a) Reconstruction of the Pr calibration phantom for each energy bin. PrONS 0.2, 0.5, 1, 2, 4, 8, 10, 20, 40, 60 mg mL^−1^, lipid and water vials loaded in MARS material phantom; Spectral response of the detector for calibration phantom across E1, E2, E3, E4 and E5 energy bins b) Linear attenuation, c) Hounsfield Unit. Linearity of attenuation of PrONS in each energy bin d) Linear attenuation e) Hounsfield Unit. The regression curves show that the SPCCT response is linear; Attenuation enhancement observed in energy bin 2 indicates the k-edge of Pr; 42.9 keV as expected. Linear relationship with R^2^ value of 0.99 enables the accuarate quantification of unknown concentrations of Pr from MD images. Data is presented as mean *±* standard deviation (*N=3*)


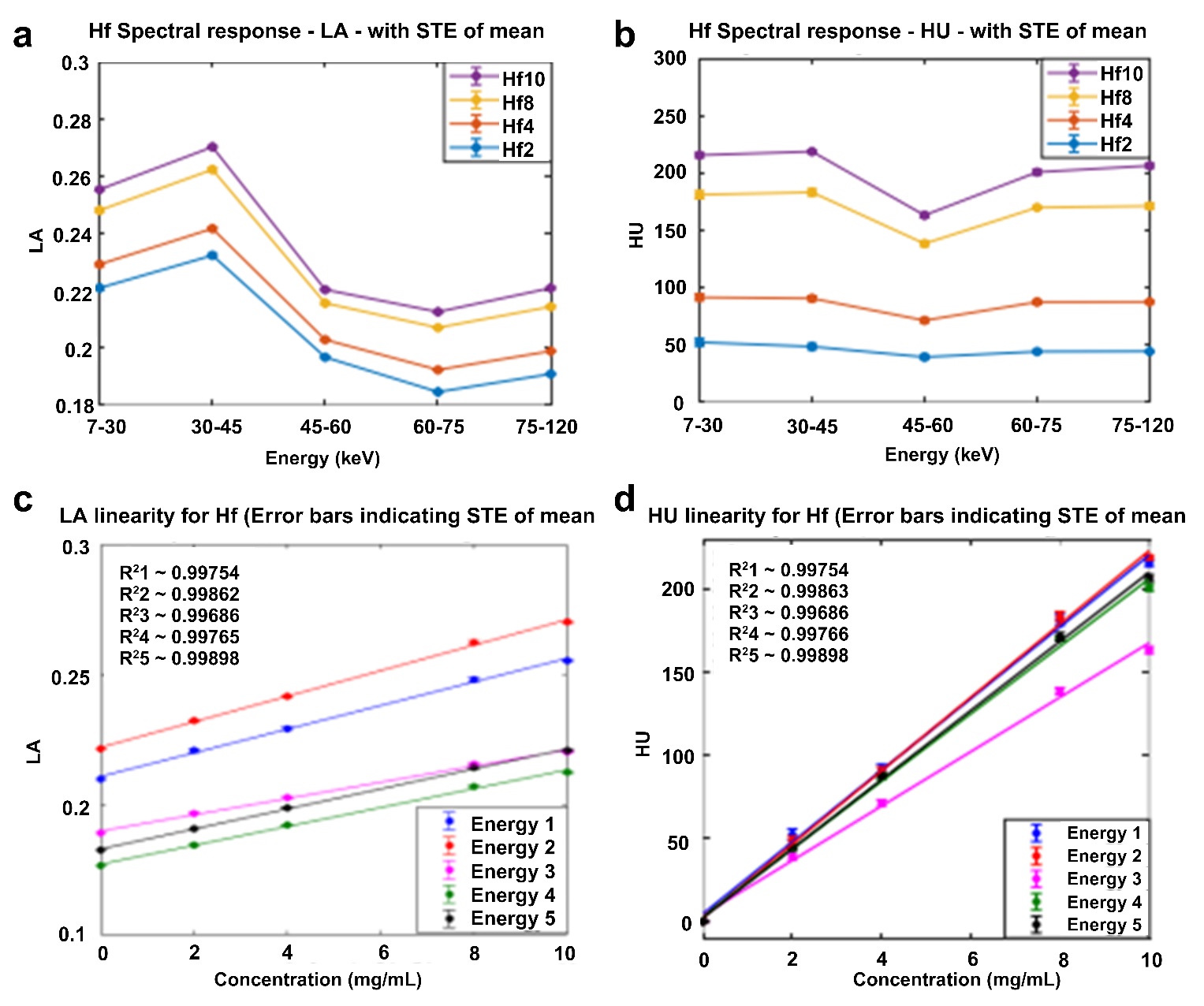


**Figure S9** Hafnium SPCCT linearity curves, Graphs indicating measured concentration using MIQ versus actual concentration for multiple concentrations of Hf, a) & b) Spectral response of the detector for Hf vials, lipid, and water (LA and HU). For Hf solutions, attenuation enhancement was observed in energy bin 4 (60-75keV), c) & d) Linearity of attenuation of Hf (LA and HU). Data is presented as mean *±* standard deviation (*N=3*)


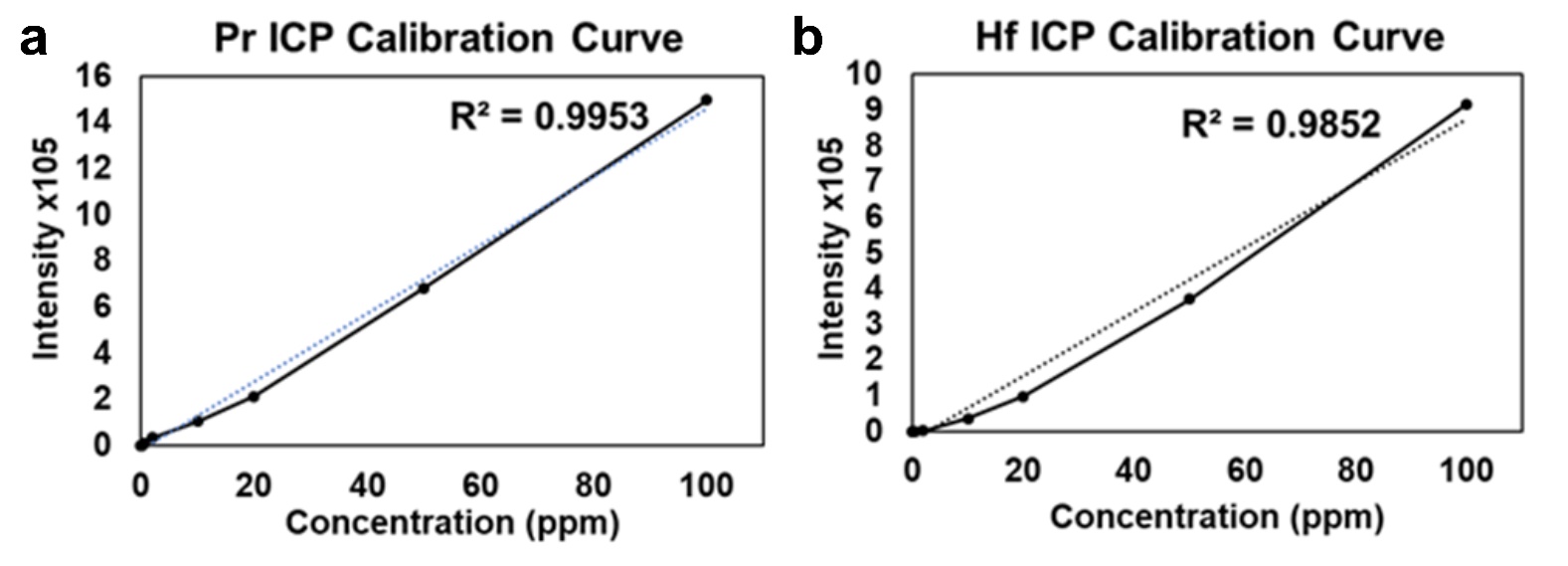


**Figure S10** ICP-OES calibration curves for PrONS and HfONS metal content quantification for comparative analysis and validation of SPCCT quantification


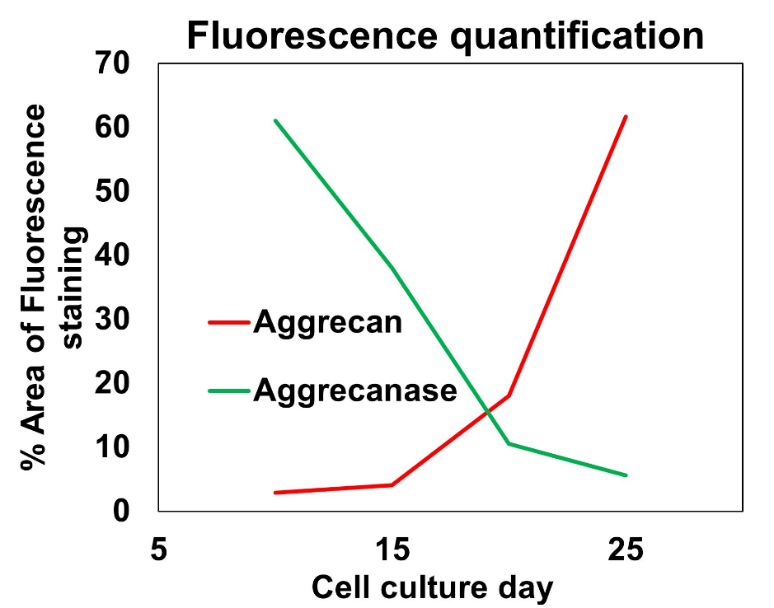


**Figure S11** Graph showing semi-quantitative % area of fluorescence staining versus cell culture day; an inverse trend in aggrecan and aggrecanase expression is observed with differential Hf/Pr particle uptake with ATDC5 multi-layer ECM growth

**Tables**

| **Sample** | **C** | **Hf** | **N** | **O** | **Pr** | **S** | **Si** | **Sample** |
| --- | --- | --- | --- | --- | --- | --- | --- | --- |
| PrONS | 39.9 | - | 2.1 | 45.9 | 5.8 | 2.2 | 4.1 | PrONS |
| HfONS | 30.1 | 11.2 | 2.7 | 51.9 | - | 1.3 | 2.7 | HfONS |

**Table S1** Concentration of elements detected (in atom%) obtained from XPS analysis for PrONS and HfONS

| **Material**  **(mg mL^-1^)** | **% Ca** | **% Lipid** | **% Pr** | **% Water** |
| --- | --- | --- | --- | --- |
| Pr 0.2 | 10.64057 | 39.28302 | 36.05087 | 90.85723 |
| Pr 0.5 | 6.661084 | 28.30438 | 46.43814 | 94.05066 |
| Pr 1.0 | 4.595981 | 11.9259 | 79.48399 | 97.99561 |
| Pr 2.0 | 1.432908 | 4.697509 | 93.76492 | 98.92297 |
| Pr 4.0 | 0.163282 | 0.24597 | 99.5897 | 99.95499 |
| Pr 8.0 | 0 | 0 | 100 | 100 |
| Pr 10.0 | 0 | 0 | 100 | 100 |
| Pr 20.0 | 0 | 0 | 100 | 100 |
| Pr 40.0 | 0 | 0 | 100 | 100 |
| Pr 60.0 | 0 | 0 | 100 | 100 |
| Lipid | 0 | 100 | 0 | 27.69939 |
| Water | 18.07934 | 35.4239 | 30.99749 | 92.34561 |

**Table S2** Material identification using MIQ algorithm for the Pr/Ca phantom. The algorithm quantifies percentage of each element (Ca, lipid, Pr, water) identified in each vial loaded into the MARS phantom showing the accuracy of detecting Pr at various concentrations

| **Parameters** |  |
| --- | --- |
| **Tube Voltage** | 118kVp |
| **Tube Current** | 20 µA |
| **Exposure Time** | 160 ms |
| **SDD, SOD** | 281.9 mm, 211.99 mm |
| **Field of View (FoV)** | 110 mm |
| **Circular projections, Flat fields** | 720 over 360°, 720 |
| **Voxel Size** | 0.09×0.09×0.09 mm3 |
| **Filtration** | 3.8 mm (2mm Al + 1.8 mm Al intrinsic) |
| **Energy bins (CSM)** | 7-30, 30-45, 45-60, 60-76, 76-118 keV |

**Table S3** Optimized MARS scanning protocol parameters for Pr detection. The energy bin thresholds were set appropriately to account for the k-edge value of Pr and to maximize signal output

| **Material**  **(mg mL-1)** | **% Ca** | **% Lipid** | **% Pr** | **% Water** |
| --- | --- | --- | --- | --- |
| Ca 35.0 | 99.97244 | 0.001901 | 0.025661 | 99.9981 |
| Ca 70.0 | 100 | 0 | 0 | 100 |
| Ca 140.0 | 100 | 0 | 0 | 100 |
| Pr 1.0 | 6.917886 | 19.42787 | 72.52424 | 97.46911 |
| Pr 2.0 | 1.905531 | 6.972059 | 91.08059 | 99.12754 |
| Pr 4.0 | 0.070329 | 0.445733 | 99.48394 | 99.91541 |
| Pr 8.0 | 0 | 0.057974 | 99.94202 | 99.99049 |
| Pr 10.0 | 0 | 0.019008 | 99.981 | 100 |
| Pr 20.0 | 0 | 0 | 100 | 100 |
| Lipid | 0 | 100 | 0 | 29.11424 |
| Water | 20.05132 | 51.64513 | 15.27466 | 91.57954 |

**Table S4** Material identification by MARS MIQ algorithm of Pr, Ca, lipid and water loaded phantom. The percent identified by MIQ for each material for every individual vial loaded in the phantom shows the accuracy of the MARS system in detecting multiple materials in a single scan. The cross talk between the materials reduces with increase in material concentrations with >90% accuracy for praseodymium at a concentration of 2 mg mL^-1^ and ~100% accuracy at 4 mg mL^-1^ concentration of Pr. Calcium has very minimal cross talk with praseodymium indicating high accuracy in separating the praseodymium-based contrast agents from bone in biological samples
